# Native/citrullinated LL37-specific T-cells help autoantibody production in Systemic Lupus Erythematosus

**DOI:** 10.1038/s41598-020-62480-3

**Published:** 2020-04-03

**Authors:** R. Lande, R. Palazzo, N. Gestermann, C. Jandus, M. Falchi, F. Spadaro, V. Riccieri, E. A. James, A. Butera, M. Boirivant, L. Feldmeyer, I. Surbeck, J. Di Lucca, F. Stuber, F. R. Spinelli, E. Botti, B. Marinari, L. Bianchi, R. Pica, B. Cerbelli, K. Giannakakis, S. E. Auteri, I. Daniels, L. G. Durrant, S. Horstman, A. Costanzo, P. Romero, C. Alessandri, F. Conti, G. Valesini, M. Gilliet, C. Chizzolini, L. Frasca

**Affiliations:** 1 Istituto Superiore di Sanità, National Centre for pre-clinical and clinical drug research and evaluation, Pharmacological research and experimental therapy Unit, 00166 Rome, Italy; 2 University Hospital CHUV, Dept. of Dermatology, 1011 Lausanne, Switzerland; 3 Translational tumor immunology group, Department of fundamental oncology, University of Lausanne, 1066 Epalinges, Switzerland; 4 Istituto Superiore di Sanità, National AIDS Center, 00166 Rome, Italy; 5 Istituto Superiore di Sanità, Confocal Microscopy Unit, Core Facilities, Rome, Italy; 6 Division of Rheumatology, Internal medicine and medical specialties, University Sapienza, Rome, Italy; 7 Department of Translational Research, Benaroya Research Institute, Seattle, WA USA; 8 Department of Systems Medicine University of Tor Vergata, Dermatology Unit, Rome, Italy; 9 “Sandro Pertini” Hospital, IBD, GE Unit, Rome, Italy; 10 Department of Radiological, oncological and anatomo-pathological sciences, Sapienza University, Rome, Italy; 11 Present Address: Department of Medico-Surgical Sciences and Biotechnologies, Sapienza University, Latina, Italy; 12 Department of Pathology, Sapienza University, Rome, Italy; 13 Academic Department of Clinical Oncology, University of Nottingham, Nottingham, UK; 14 Skin pathology laboratory, Dermatology Unit, IRCCS Humanitas clinical and research center, Rozzano, Milan, Italy; 15 Department of Immunology and Allergy, University Hospital and Medical School, Geneva, Switzerland

**Keywords:** Immunology, Adaptive immunity, Autoimmunity

## Abstract

LL37 exerts a dual pathogenic role in psoriasis. Bound to self-DNA/RNA, LL37 licenses autoreactivity by stimulating plasmacytoid dendritic cells-(pDCs)-Type I interferon (IFN-I) and acts as autoantigen for pathogenic Th17-cells. In systemic lupus erythematosus (SLE), LL37 also triggers IFN-I in pDCs and is target of pathogenic autoantibodies. However, whether LL37 activates T-cells in SLE and how the latter differ from psoriasis LL37-specific T-cells is unknown. Here we found that 45% SLE patients had circulating T-cells strongly responding to LL37, which correlate with anti-LL37 antibodies/disease activity. In contrast to psoriatic Th17-cells, these LL37-specific SLE T-cells displayed a T-follicular helper-(T_FH_)-like phenotype, with CXCR5/Bcl-6 and IL-21 expression, implicating a role in stimulation of pathogenic autoantibodies. Accordingly, SLE LL37-specific T-cells promoted B-cell secretion of pathogenic anti-LL37 antibodies *in vitro*. Importantly, we identified abundant citrullinated LL37 (cit-LL37) in SLE tissues (skin and kidney) and observed very pronounced reactivity of LL37-specific SLE T-cells to cit-LL37, compared to native-LL37, which was much more occasional in psoriasis. Thus, in SLE, we identified LL37-specific T-cells with a distinct functional specialization and antigenic specificity. This suggests that autoantigenic specificity is independent from the nature of the autoantigen, but rather relies on the disease-specific milieu driving T-cell subset polarization and autoantigen modifications.

## Introduction

Systemic Lupus Erythematosus (SLE) is an autoimmune disease with a prevalence of about 20–50 cases per 100,000 in Northern Europe and USA^1^, characterized by immune-complex formation and deposition, which result in inflammation and tissue damage. In SLE, altered clearance of dying cells determines persistent exposure of autoantigens and activation of antigen-presenting cells (APCs), mainly *via* Toll-like receptors (TLR), thus favoring adaptive immune response licensing^1–3^. SLE autoantibodies are preferentially directed against nuclear antigens (ANA)^1–3^, but in a substantial subset of SLE patients, antibodies target cytoplasmic proteins in neutrophils^4,5^. Neutrophils are indeed crucial players in SLE pathogenesis^5,6^. Pathways linked to neutrophil activation (apoptosis, neutrophil-extracellular-trap release, NET, extrusion of oxidized DNA by living cells) were believed implicated in autoimmune B-cell activation^5–7^. Although life expectancy has greatly improved, SLE remains a devastating disease with a standardized mortality ratio in excess of three^8^, thus novel therapeutic strategies and new therapeutic targets are necessary^1,2^. We have discovered new autoantibody specificities and key self-proteins of the group of antimicrobial peptides (AMPs), which could drive pathogenic events in SLE^9^. LL37 is of particular interest, since it binds and protects self-nucleic acids from degradation, forming complexes that prolong DNA/RNA half-life. These LL37-nucleic acid complexes act as danger-associated molecular patterns (DAMPs)^9–12^. Complexes formed by LL37 with DNA/RNA elicit the production of type I interferon (IFN-I) and other pro-inflammatory cytokines by plasmacytoid, myeloid dendritic cells (pDCs, mDCs), and monocytes^9–12^. Anti-LL37 antibodies favor the up-take of LL37-DNA complexes into DCs^9^, which enhances IFN-I production. Finally, LL37-DNA complexes can directly stimulate B-cells to produce antibodies, including anti-LL37 antibodies^13^. Since neutrophils accumulate in SLE target organs, including skin and kidneys, and are important releasers of LL37, we anticipate that increased LL37 concentration and LL37 binding to self-DNA/RNA, coupled to generation of anti-LL37/LL37/DNA/RNA immune-complexes, would concur to pathogenic events in SLE^14–16^. Notably, in psoriasis LL37 frequently acts as T-cell autoantigens^17^. We hypothesized that LL37 is highly immunogenic for T-cells because its sequence contains multiple binding motifs for several HLA-class I/II alleles. However, it is presently unclear whether: (a) anti-LL37 antibodies correlate with disease activity; (2) LL37-DNA complexes and/or NET/NET-like structures are present in lupus-target tissues; (3) T-cells responding to LL37 exist and correlate with, and/or participate to, SLE pathogenesis. The latter aspect is important in that T-cells with T-helper follicular (T_FH_) functions are necessary for isotype immunoglobulin (Ig)-switch and somatic hypermutation, the processes that generate high affinity autoantibodies^17–20^.

By analyzing distinct patient cohorts, here we demonstrate that high anti-LL37 antibody levels specifically circulate in SLE and not in psoriasis or control chronic diseases, and correlate with disease activity. LL37 and citrullinated LL37 (cit-LL37) are present in SLE skin/kidney, and SLE T-cells proliferate to both native-LL37 and cit-LL37 in up to 45% of SLE patients, with cit-LL37 being a more efficient T-cell antigen than native-LL37. SLE T-cell responses significantly correlate with anti-LL37 antibodies and disease activity, suggesting that LL37-directed responses are markers of active/severe SLE. Notably, unlike autoreactive psoriasis T-cells, SLE native-LL37/cit-LL37-specific T-cells often possess a T-follicular helper (T_FH_)-like phenotype, and drive the production of pathogenic anti-LL37/anti-DNA/RNA antibodies *in vitro*. Thus, the efficient dual immune amplification role of LL37 on both innate and adaptive immunity, favoured by the LL37 adjuvant activity and immunogenicity, operates also in SLE. However, the intrinsic properties of this antigen, although efficacious in both settings, lead to different outcomes, dictated by the disease-specific milieu.

## Results

### LL37, cit-LL37 and LL37-DNA complexes are present in lupus target organs

**Figure 1:**
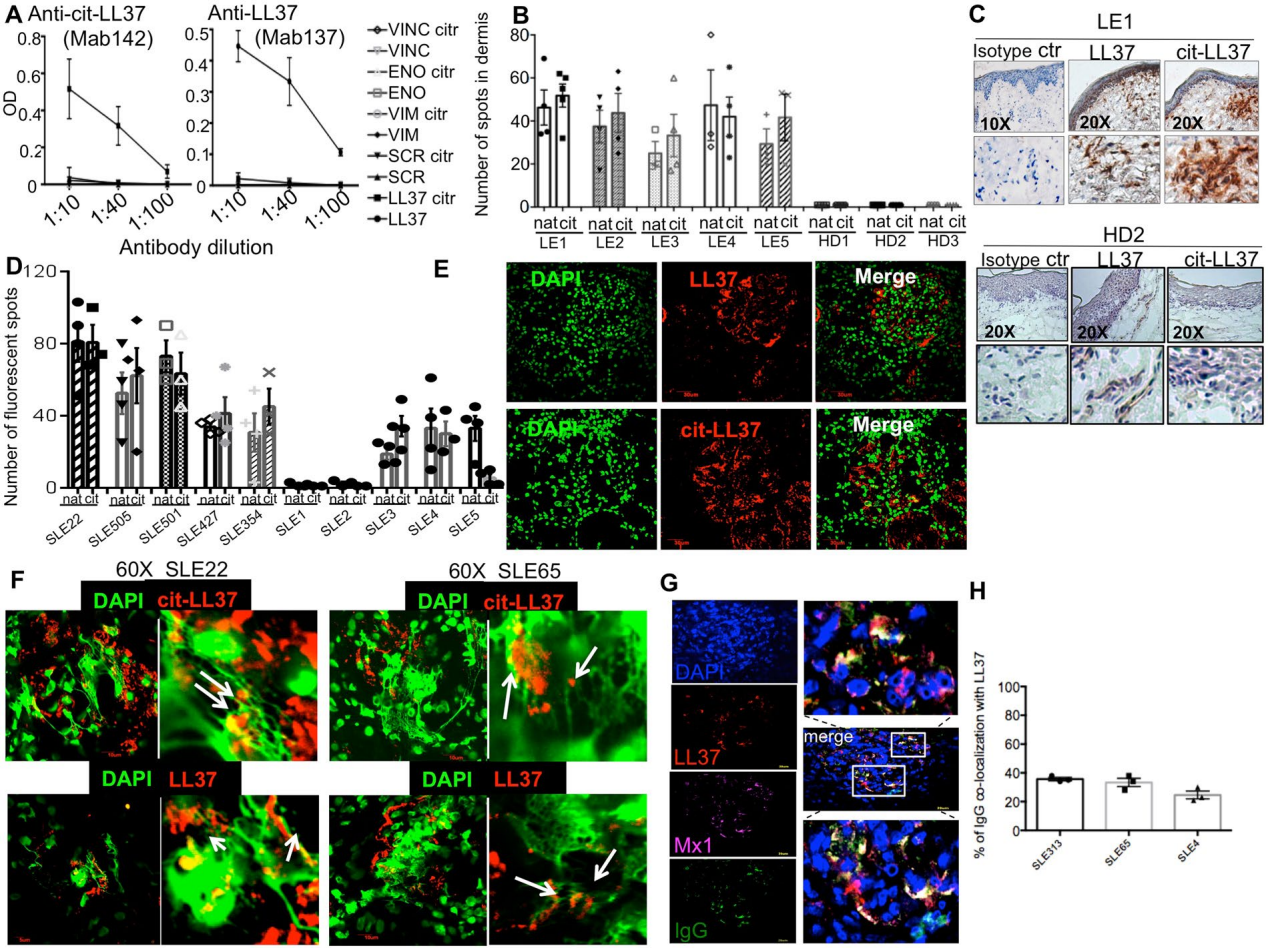
LL37, cit-LL37- and LL37-DNA complexes are present in SLE affected tissues. (**A**) Dose response reactivity (expressed as Optical Density, OD) of Mab142 and Mab139, to LL37, cit-LL37 and control peptides (vimentin, VIM, eolase, ENO, vinculin, VINC, scramble LL37, SCR, in their native or citrullinated (citr) forms. (**B**) Number of LL37-positive and cit-LL37 positive spots in CLE and HD dermis assessed by immunohistochemistry. For quantification at least 3 sections for each patients (n = 5) and HD (N = 3) were assessed. Data represent mean ± SE. (**C**) Representative image of immunoistochemistry of the skin of one CLE, upper panels or one HD, lower panels, assessed for expression of native LL37 or cit-LL37. (**D**) Quantification of LL37 and cit-LL37 in 10 SLE-affected kidney biopsies. For quantification at least 3 sections for each patients (n = 10) were assessed. Data represent mean ± SE. (**E**) representative confocal images of staining of SLE kidney biopsies assessed for presence of LL37 or cit-LL37 (magnification 600×). (**F**) Representative confocal images of two out of seven SLE affected renal biopsies showing expression of native or cit-LL37 and DNA filaments (DAPI). (**G**) Confocal images showing expression of LL37, immune-complexes (IgG) and the IFN-induced Mx1. The panels on the left show magnification of two areas to show colocalization (yellow) of IgG staining with LL37 staining. (**H**) Quantification (as percentage) of co-localization between IgG staining and LL37 staining, in SLE renal biopsies from three different patients. For quantification 3 sections for each patient were assessed. Data represent mean ± SE. All biopsies were from lupus nephritis (class IV). Skin biopsies were form CLE patients.

LL37 is produced by activated neutrophils and is abundantly released in neutrophil-extracellular-like (NET)-structures^9^. We previously hypothesized that neutrophils release high amounts of LL37 and DNA in SLE tissues, favoring IFN-I production and the activation of inflammatory pathways, which would fuel autoimmunity^9^. Thus, here we have searched for LL37 and LL37-DNA complexes in SLE-target tissues. Moreover, since peptidyl arginine deaminase (PAD) enzymes can become activated during inflammation^21–23^ and perform citrullination of arginine-rich proteins, we specifically searched for the presence of cit-LL37 in SLE/cutaneous lupus (CLE) tissues. To do so, we took advantage of specially developed monoclonal antibodies directed to either native LL37 (Mab137) or cit-LL37 (Mab142), which lack cross-reactivity between the two LL37 forms and to unrelated control proteins/peptides (in their native and citrullinated forms, Fig. 1A). We detected both native-LL37 and cit-LL37 in SLE and CLE target organs by immunohistochemistry (Fig. 1B,C) and laser scanner confocal microscopy (LSM) (Fig. 1D,E). Quantification, of native LL37 and cit-LL37 showed that the two LL37 forms were mostly present in the skin (dermis) and the kidney sections in comparable amounts (Fig. 1B,D, respectively) (see Suppl. Methods). Of note, healthy donor (HD) skin did not express significant amounts of either LL37 forms, as expected, as HD skin does not usually have a neutrophil infiltrate (Fig. 1B,C, lower panels). In addition, we clearly detected LL37 complexed with DNA in the kidneys of patients with lupus nephritis (Fig. 1F). Within four of seven kidney biopsies analyzed (57%), DNA appeared in the form of filaments bound to LL37 and/or cit-LL37 (Fig. 1F). LL37, and LL37-DNA complexes co-localized with, or were in the proximity of, the interferon-induced GTP-binding protein Mx1 (Mx1), an IFN-I-activated gene product^24^, and immuno-globulin (IgG) deposition (Fig. 1G, Fig. S1). An estimation of the percent of IgG staining which co-localizes with LL37 staining is shown in Fig. 1H (see also Suppl. Methods). Since complement deposition in the form of membrane-attack-complexes (MAC) can be responsible for hypercitrullination of multiple substrates in other settings^25,26^, we assessed the simultaneous presence of cit-LL37 and complement C9 deposition, which indeed could co-localize in SLE kidney (Fig. S2).

These results suggest that LL37 forms complexes with self-DNA and autoantibodies in SLE-affected tissues, which can enable LL37 to act as an adjuvant and exert local interferogenic activity, revealed by the expression of Mx1. The results also demonstrate that a consistent part of LL37 undergoes citrullination in SLE-affected tissues.

### Anti-native LL37 and anti-cit-LL37-specific antibody reactivity correlates with SLEDAI and declines in inactive SLE

**Figure 2:**
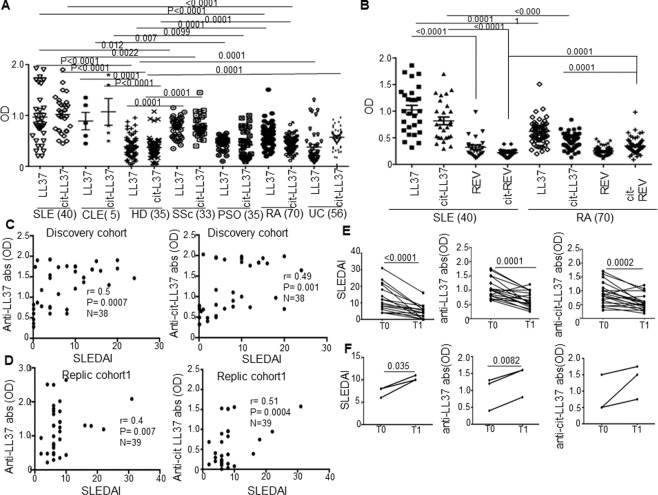
Anti-native LL37/cit-LL37 antibodies correlate with disease activity. (**A**,**B**) Antibody reactivity of SLE to native LL37/cit-LL37 (**A**), compared to that of control HD, SSc, PSO, RA and UC sera, measured by ELISA. (**B**) Antibody reactivity, by ELISA (OD), of SLE and RA sera to LL37/cit-LL37, as compared to control REV and citrullinated REV peptides. In A and B, horizontal bars represent the mean, vertical bars standard errors of the mean, *P*-values by Student’s t-test for paired samples to compare anti-native LL37/cit-LL37 response in the same patient, for unpaired samples for inter-group comparison; number of individuals tested indicated. (**C**,**D**) Anti-native LL37/anti-cit-LL37 antibody response (OD) plotted against SLEDAI. Spearman’s r, significance P, sample size N, indicated. (**E**,**F)** SLEDAI decrease (**E**) or increase (**F**) between T0 and T1 in 21 (**E**) and 3 (**F**) LL37-responder SLE patients, responding **(E)**/non responding **(F)** to therapy and concomitant decline (**E**)/increase (**F**) of anti-native LL37/anti-cit-LL37 antibody reactivity (OD); P-values by Student’s t-test for paired samples.

We have shown previously that a consistent proportion of SLE patients harbor circulating anti-LL37 antibodies^9,13^, as compared to HD. In this study, anti-LL37 antibody reactivity correlated with SLE severity^9^. A second study reported the presence of circulating anti-LL37 antibodies in SLE but did not confirm a correlation with disease activity^27^. Thus, we assessed the presence of anti-LL37 antibodies in SLE cohorts (Table S1), and a small group of CLE patients^28^ (Table S2). Sera from SSc, RA, UC and psoriasis patients, diseases in which LL37 is hyper-expressed in tissues, and HD, served as controls^10,29–33^. In addition, we searched for anti-cit-LL37 antibodies. Twenty-one (53%) and eighteen (48%) of 40 SLE patients had detectable and significant antibody reactivity to native LL37 and cit-LL37, respectively (Fig. 2A). Compared to HD, we observed a weaker and/or occasional reactivity to native LL37/cit-LL37 in control psoriasis psoriasis, RA, SSc and UC patients, which was significantly weaker when compared to SLE reactivity. Most importantly, antibody responses to control reverse LL37 (REV) and cit-REV peptide were negative in SLE, which suggests that circulating SLE antibodies truly recognized cit-LL37 and not merely citrulline (Fig. 2B). In contrast, reactivity to cit-REV was detected in a significant proportion of RA sera (14 of 70 RA, 20%), which is consistent with the reported capacity of RA patients to mount a broad antibody response to citrullinated proteins^23^ (Fig. 2B). Most importantly, anti-LL37 and anti-cit-LL37 antibodies correlated with disease activity only in SLE patients (using Systemic Lupus Erythematosus Disease Activity Index, SLEDAI, see Methods). We observed no correlation with disease activity (see Methods), in control disease patients, [for psoriasis, psoriasis-activity index, PASI: Spearman’s r (a-LL37) = −0.31 N = 33, p = 0.31; r (a-cit-LL37) = −0.34, N = 33, P = 0.32]; for RA [DAS28: Spearman’s r (a-LL37) = −0.06 N = 40, p = 0.9; r (a-cit-LL37) = 0.07, N = 40, P = 0.69]; for UC [Mayo endoscopic Score, Spearman’s r (a-LL37) = −0.05 N = 58, p = 1.1; r (a-cit-LL37) = 0,002, N = 58, P = 1.0]; for SSc [Rodnan skin score: Spearman’s r (a-LL37) = −0.21 N = 33, P = 0.1; r (a-cit-LL37) = 0,0008, N = 33, P = 0.99]. We confirmed SLE correlations data in a replication cohort (Replication cohort1, See Table S1, Fig. 2D). Anti-LL37 antibodies also correlated with serum IFN-α in two patients cohorts (discovery cohort: Spearman r = 0.46, p = 0.0019, N = 38, for anti-LL37 abs, r = 0.3, p = 0.035, N = 3 for anti-cit-LL37 abs; replication cohort2: r = 0.42, p = 0.04, N = 18 for anti-LL37 abs, r = 0.5, p = 0.016, N = 18, N = 18 for anti-cit-LL37 abs).

To observe how antibody reactivity behaved during the course of disease, we selected twenty-four patients with active disease (SLEDAI > 4, range 4-to-32; mean SLEDAI = 12.6 ± 7.5), which were positive for anti-native LL37 and anti-cit-LL37 antibodies at first sampling. During the following 6-to-36 months, the SLEDAI decreased significantly in twenty-one (88%) (Fig. 2E, left panel) but not in three of them (12%, Fig. 2F, left panel) in response to treatment (Plaquenil, prednisone, See also Table S1). In the LL37-responder patients, anti-native LL37 and anti-cit-LL37 antibody reactivity declined in parallel with SLEDAI reduction (Fig. 2E, right panels). In contrast, in the three unresponsive patients (Fig. 2F, right panels) anti-native LL37 and anti-cit-LL37 antibody reactivity increased.

These results show that when a significant anti-LL37 humoral response is specifically present in SLE, this correlates with disease activity and declines with it, suggesting a role of antibody reactivity to LL37 and cit-LL37 as disease markers and, possibly, involvement in disease pathogenesis.

### T-cells respond to LL37 and cit-LL37 in SLE and their responses correlate with SLEDAI and anti-LL37/anti-cit-LL37 antibodies

**Figure 3:**
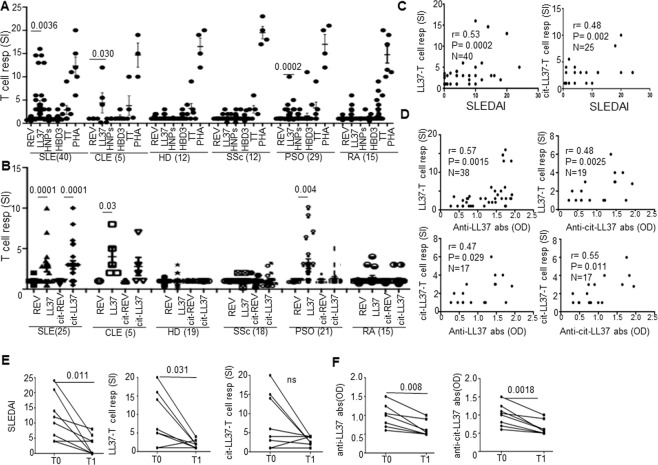
Circulating T-cells of SLE/CLE patients proliferate to LL37/cit-LL37. (**A**,**B**) Cumulative data of proliferative response of SLE/CLE patients, HD, and control SSc, PSO and RA patients to LL37, control AMPs (HBD3 and HNPs) and PHA (**A**), and to LL37, cit-LL37 and relative REV control peptides (**B**), expressed as stimulation indexes (SI), defined as % of BrdU-incorporation of peptide-stimulated cells over % of BrdU-incorporation of untreated cells (33). Number of individuals tested indicated. Values of SI > 3 were considered positive (See Methods). Horizontal bars represent the mean, vertical bars are standard errors of the mean, *P*-values by Student’s t-test for paired samples. (**C**,**D**) LL37-specific T-cell proliferation (SI) plotted against disease activity, SLEDAI (**C**) and against anti-LL37/cit-LL37 antibody reactivity (OD). Spearman’s coefficient r, significance P, sample size N, indicated. (**E**) SLEDAI decrease between T0 and T1 in 8 SLE patients responding to therapy (Table S1) and concomitant decline of T-cell reactivity (SI) to native LL37/cit-LL37 (SI), and (**F**) decline of antibody reactivity as at the same time points. P-values from Wilcoxon signed-rank test.

A previous *in silico* analysis identified promiscuous T-cell epitopes for multiple HLA-DR alleles in the LL37 sequence, explaining why LL37 is very likely to be immunogenic for T-cells, an assumption verified in psoriasis^17^. Thus, given the consistent expression of LL37 in SLE tissues, and the LL37 ability to form LL37-DNA complexes *in vivo*, we tested whether LL37 behaves as a T-cell autoantigen also in SLE/CLE. SLE/CLE blood mononuclear cells (PBMCs) were cultured with LL37 (Fig. S3, for gating strategy) or control unrelated AMPs human β-defensin-3 (HBD3) and α-defensins HNP_1–3_, or with tetanus toxoid (TT) and phytohemagglutin (PHA), as positive controls. At day 6, we assessed BrdU-incorporation in CD3+CD4^+^ T-cells and considered T-cell responses positive those with stimulation index was ≥3 (see Methods)^17^. Cumulative data in Fig. 3A show that CD3+CD4+ cells from 18 of 40 SLE (45%) (and from three of five CLE patients), proliferated in response to LL37, whereas responses to HBD3 or HNP_1–3_ were not significant (Fig. 3A). No SSc or HD showed a significant response to LL37 and other AMPs, whereas ten of twenty-nine PSO patients (34%) responded in a significant manner, as expected^17^. T-cells from two out of 15 (13%) RA patients proliferated to LL37. We observed proliferation to cit-LL37 in 11 of 25 (44%) SLE and 3 of 5 CLE patients but we observed no responses in HD (Fig. 3B). T-cells from two of 15 RA (13%) and 3 of 21 PSO (14%) patients also responded to cit-LL37, suggesting that cit-LL37 can be occasionally immunogenic also in psoriasis and RA. In these assays, the control reverse LL37 peptide (REV, Table S2) did not elicit T-cell responses, ensuring that T-cell activation by LL37 was sequence dependent and independent of its DNA binding ability^9,17^. Indeed, the REV peptide has an inverse LL37 sequence (REV), but retains DNA-binding activity^17^. Moreover, we also observed T-cell proliferation (Fig. S4A) to shorter (13–15mer) overlapping LL37-peptides (Table S2, Fig. S4A) and to cit-LL37, all completely devoid of adjuvant activity for DCs, as shown in Fig. S4B. When we plotted the values of LL37/cit-LL37 induced T-cell proliferation against disease activity as assessed by SLEDAI, we found a positive correlation (Fig. 3C). We also correlated the same parameters to “clinical” SLEDAI (without inclusion of data regarding presence/absence of anti-DNA antibodies, or complement reduction) and we found similar correlations (T-cell response to LL37 versus clinical SLEDAI: r = 0.52, P = 0.0007, N = 40; T-cell responses to cit-LL37 versus clinical SLEDAI: r = 0.49, p = 0.02, N = 24). Interestingly, T-cell responses to both native LL37 and cit-LL37 were more frequent in SLE patients with malar rash according to Chi-square test (z = 1.84, P = 0.033, N = 25, for response to LL37 and z = 1.7, P = 0.047, N = 25 for response to cit-LL37). Moreover, the magnitude of the T-cell responses (as SI) was higher in SLE patients with malar rash than in patients with no malar rash (Mann-Whitney test: P = 0.039, N = 25, for response to native LL37 and P = 0.017, N = 25, for response to cit-LL37) and in patients with renal involvement, as compared to patients without renal involvement (Mann-Whitney test: P = 0.045, N = 30 for response to native LL37, and P = 0.034, N = 25, for response to cit-LL37). Of note, the magnitude of T-cell response to LL37 (but not to cit-LL37) was also higher in patients with anti-DNA antibody positivity, than in patients with anti-DNA antibody negativity (Mann-Whitney test: P = 0.0078, N = 17). In psoriasis patients we confirmed a positive and significant correlation between T-cell response to LL37 and PASI (Spearman’s r = 0.33, p = 0.04, N = 29)^17^, whereas T-cell responses to LL37/cit-LL37 in RA did not correlate with disease activity (expressed as DAS28) (Spearman’s r = 0.07, p = 0.34, N = 38, for both responses). Most importantly, T-cell responses to native LL37/cit-LL37 correlated with the presence of autoantibodies to both native LL37 and cit-LL37, in SLE (Fig. 3D). These data indicate that LL37 can act as an autoantigen for SLE CD4 T-cells in both its native and citrullinated form. Eight patients, that at first sampling had T-cell proliferating to LL37, were available for longitudinal follow-up and were re-tested for T-cell reactivity to LL37/cit-LL37 (after up to 36 months of successful standard therapy, as above). Their T-cell responses decreased (and this decrease was statistically significant for proliferation to native LL37). Reduction in magnitude of the responses coincided with a reduction in SLEDAI (Fig. 3E). Of interest, in the same patients, the antibody reactivity to native LL37 and cit-LL37 significantly declined in parallel (Fig. 3F). These studies provide correlative evidence for the participation of autoreactive native-LL37/cit-LL37-specific T-cells to LL37 antibody production and disease activity. Moreover, T-cell responses to native LL37 and cit-LL37 may behave as biomarkers in SLE, at least in the LL37-responder patients.

### LL37/cit-LL37-specific T-cells sustain the production of pathogenic anti-native LL37, and anti-DNA/RNA antibodies

**Figure 4:**
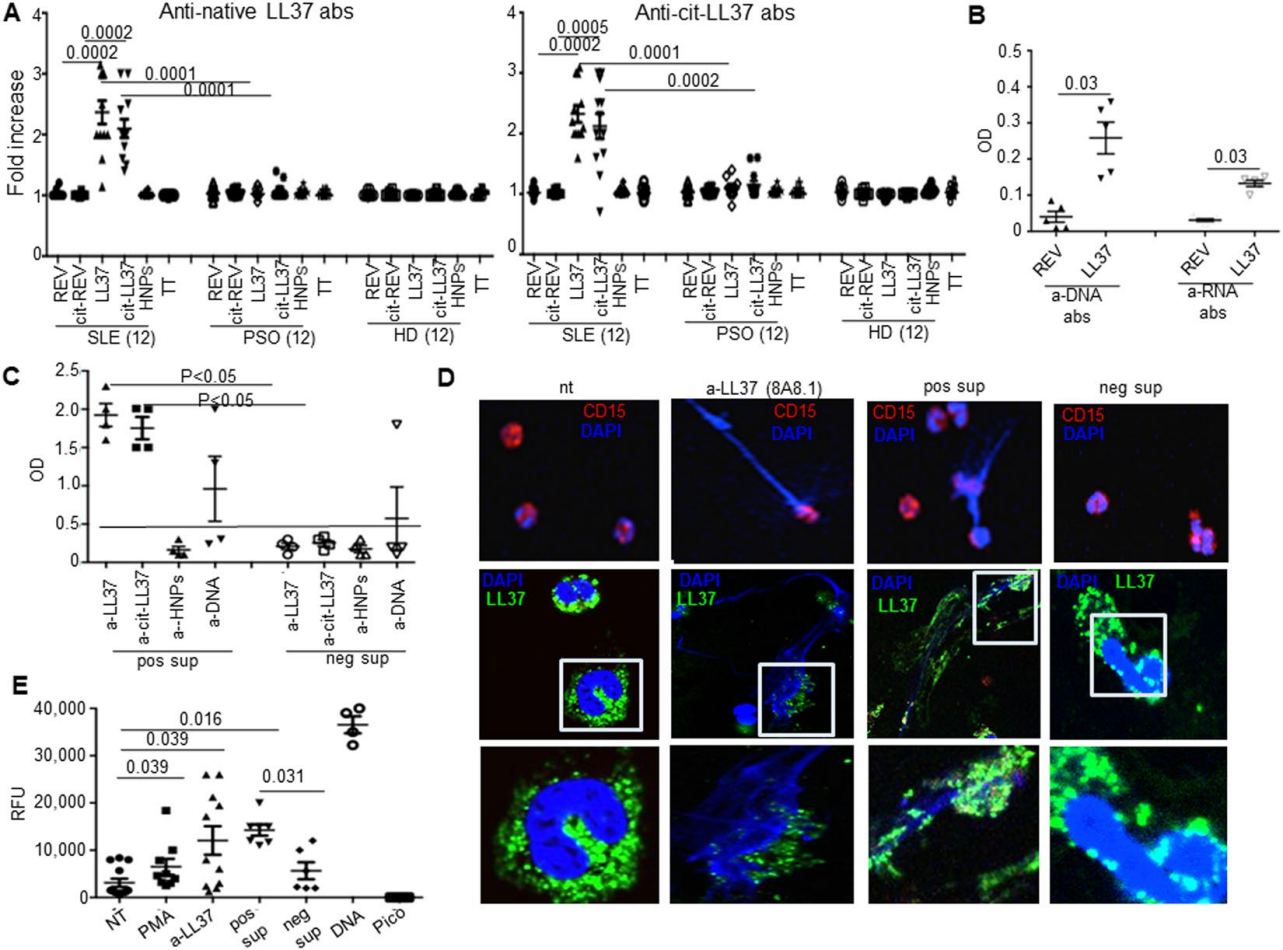
Anti-LL37 T-cells drive production of pathogenic anti-native LL37/cit-LL37 antibodies **an**. (**A**) SLE/PSO/HD PBMCs were stimulated for 7 days with the indicated antigens, and anti-native LL37(left)/anti-cit-LL37(right)-antibody reactivity tested by ELISA in supernatants. Sample size indicated. The results are presented as antibody fold increase of OD values with respect to untreated cells. (**B**) SLE PBMCs were stimulated twice with LL37/REV and anti-DNA antibodies analyzed by ELISA. (**C**) Immortalized B-LCLs from SLE/CLE bulk-cultures, obtained with native-LL37/cit-LL37, were tested for anti-LL37/anti-cit-LL37/anti-DNA antibody reactivity by ELISA. (**D**) SLE neutrophils were treated as indicated and stained for DNA (DAPI, blue), CD15 (red) (upper panels) or for LL37 (green) (middle panels, insets in the lower panels). Results from one representative experiment of four performed with different SLE neutrophils preparations. (**E**) SLE neutrophils were untreated (nt), or treated with the indicated stimuli; and released DNA was evaluated by PicoGreen assay (9). Results as relative fluorescence units (RFU) plus/minus standard error of the mean from five-to-ten independent experiments with different donors. RFU values of DNA/PicoGreen alone (Pico) reported. In all panels: Horizontal bars = means, vertical bars = standard errors of the mean, P values by Student’s t-test for paired samples (intragroup comparison)/unpaired (SLE vs HD or psoriasis cultures) samples.

We then assessed the helper T-cell ability for autoantibodies production of SLE LL37-responder T-cells^34^. As shown in Fig. 4A, when we cultured SLE PBMCs for 7 days in the presence of LL37 or cit-LL37 or control antigens, anti-LL37 (left panel) and anti-cit-LL37 (right panel) antibodies became apparent in culture supernatants of PBMCs stimulated with LL37 and cit-LL37, (but not in cultures stimulated with control peptides). Noteworthy, culture of PBMCs of psoriasis patients whose T-cells proliferate to LL37, did not lead to significant anti-LL37/cit-LL37 antibody production, as for HD PBMCs, suggesting that psoriasis LL37-specific T-cells did not primarily work to favor autoantibody production *in vivo*. Moreover, unrelated antibody specificities, such as anti-HNPs and anti-TT antibodies, did not increase in SLE cultures stimulated by native LL37 or cit-LL37 (Fig. S5), thus T-cell help was specific for anti-LL37 antibody production. Interestingly, upon sequential stimulation with autologous PBMCs and native LL37, both an anti-DNA- and RNA-antibody reactivity became also detectable in SLE cultures (Fig. 4B). Immortalization by Epstein Barr Virus (EBV, 33) of the SLE/CLE B-cells expanded by LL37 stimulation, generated B-LCLs, some of which produced anti-native LL37/cit-LL37 antibodies and/or anti-DNA antibodies (Fig. 4C), and similar amounts of total IgG (positive supernatants 125 ±  21 pg/mL versus negative supernatants 145 ±  16 pg/mL, p = 0.1, N = 4, Student’s t-test for unpaired samples). These anti-LL37 containing supernatants, but not anti-LL37-negative supernatants, induced extrusion of NET-like filaments decorated by LL37 (Fig. 4D,E) by SLE neutrophils *in vitro*. Thus, cit-LL37-specific T-cells (and not only native-LL37 specific T-cells), sustain the production of anti-LL37 antibodies, with consequent SLE neutrophils release of interferogenic LL37-DNA complexes.

### Circulating SLE LL37/cit-LL37-specific T-cells can express a T_FH_-like phenotype

**Figure 5:**
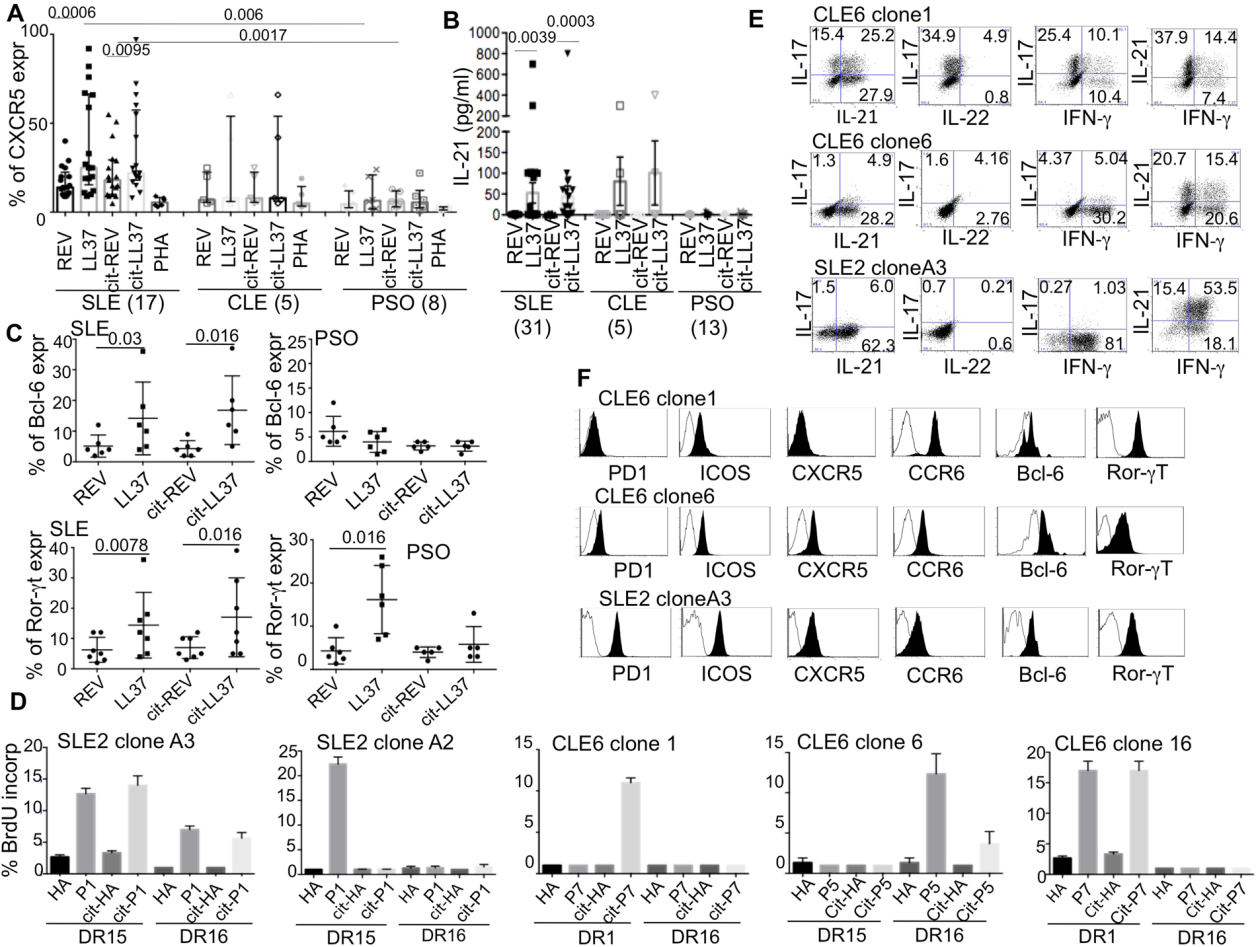
LL37-specific T-cells are T_FH-_like cells. (**A**) CXCR5 expression (see Fig. S4A–C) by BrdU^+^CD4^+^T-cells responding to the indicated stimuli. Results are shown as medians of percent of expression measured by flow cytometry, with Interquartile range (IQR). P values calculated by two-tailed Wilcoxon signed-rank test for intra-group comparison, and by Mann-Whitney test for inter group comparison. (**B**) IL-21 (pg/mL/1 × 10^6^ cells) measured by ELISA in SLE/CLE/PSO-PBMC-cultures stimulated with LL37/cit-LL37/control antigens (sample size indicated). P values by two-tailed Wilcoxon signed-rank test. (**C**) Cumulative data of Bcl-6/Ror-γt expression by flow cytometry of 48 hours stimulated PBMC (see Fig. S6D). Horizontal bars = means, vertical bars = standard errors of the mean, P-values by two-tailed Wilcoxon signed-rank test. (**D**) SLE/CLE CD4^+^ T-cell clones were cultured for three days with irradiated B-LCLs (expressing given HLA-DR) pulsed with native-LL37/cit-LL37-derived peptides (0.1-to-1 μg/mL). Percent of BrdU^+^ T-cells by flow cytometry, shown as histograms (plus/minus standard error of the mean of triplicate cultures) from one of three experiments for each T-cell clone. (**E**) LL37-specific T-cell clones, stimulated with PMA + Iono, were tested for cytokine expression by flow cytometry (shown dot plots of gated CD3^+^CD4^+^ cells). **(F)** LL37-specific T-cell clones stained for surface markers or Bcl-6/Ror-γt (intracellular staining). Expression markers (gate on CD3^+^CD4^+^ cells, black) over isotype antibody control staining (white) are shown as flow cytometry histograms. Data are from one of three representative experiments for each clone.

While CD3+CD4+ T cells proliferated in response to LL37 and cit-LL37 in both SLE and psoriasis, only in SLE a substantial humoral anti-LL37/cit-LL37 response developed^9,17,34,35^(Fig. 2), suggestive of a preferential expansion of T_FH_ in SLE. To verify this hypothesis, we assessed the expression of T_FH_ markers in PBMCs cultured in the presence of LL37/cit-LL37 in SLE or CLE, and in control psoriasis patients. In SLE (but not in psoriasis), we observed a significant up-regulation of CXCR5 (cumulative data in Fig. 5A; gating strategy in Fig. S6A–C) and IL-21 production (Fig. 5B) by LL37/cit-LL37 proliferating T-cells. Of note, secreted IL-21 levels correlated with the magnitude of LL37/cit-LL37-induced T-cell proliferation (for LL37: Spearman r = 0.64, p = 0.0001, N = 31; for cit-LL37: r = 0.5, P = 0.0013, N = 24). To avoid bias due to prolong *in vitro* culture, we assessed transcription factors expression at shorter time points (48 hours), in a limited number of patients. While both SLE and psoriasis activated (CD38^hi^,^36^) T-cells could upregulate Ror-ɣt expression^37^, only CD38^hi^ SLE T-cells, but not CD38^hi^ psoriasis T-cells, could up-regulate Bcl-6, upon stimulation with LL37/cit-LL37 (Fig. 5C) (see Fig. S6D for gating strategy).

For the unambiguous identification of LL37/cit-LL37-specific T-cells, we cloned peripheral T-cells from one SLE and one CLE patient. We obtained several T-cell clones for which we report the HLA-restriction and the fine epitope mapping in Fig. 5D. Some of the T-cell clones proliferated exclusively after stimulation with native LL37, or exclusively to cit-LL37-derived epitopes. Other clones proliferated to both native LL37 and cit-LL37 (Fig. 5D), but did not cross-react to the control peptide HA306–320, a promiscuously presented peptide derived from the hemoagglutinin protein of *Influenza* A (in both its native and citrullinated form). Th17 or T_FH_-like attributes were also clonally distributed. For instance, CLE6 clone 6 and SLE2 clone A3 produced the largest amounts of IL-21 (Fig. 5E) and expressed the T_FH_ markers CXCR5, ICOS, PD1 and Bcl-6, and low level of Ror-ɣt (Fig. 5F). CLE6 clone 1, was higher producer of IL-17 (Fig. 5E) and showed the highest expression level of CCR6 and Ror-ɣt, with modest Bcl-6 expression (Fig. 5F).

Altogether, these results show that in SLE, but not in psoriasis, at least part of the native-LL37 or cit-LL37-specific T-cells belong to the T_FH_- like subset, whereas Th17-types of cells were present in both pathologies.

### Cit-LL37 has stronger agonist activity compared to native-LL37 for T-cell activation

**Figure 6:**
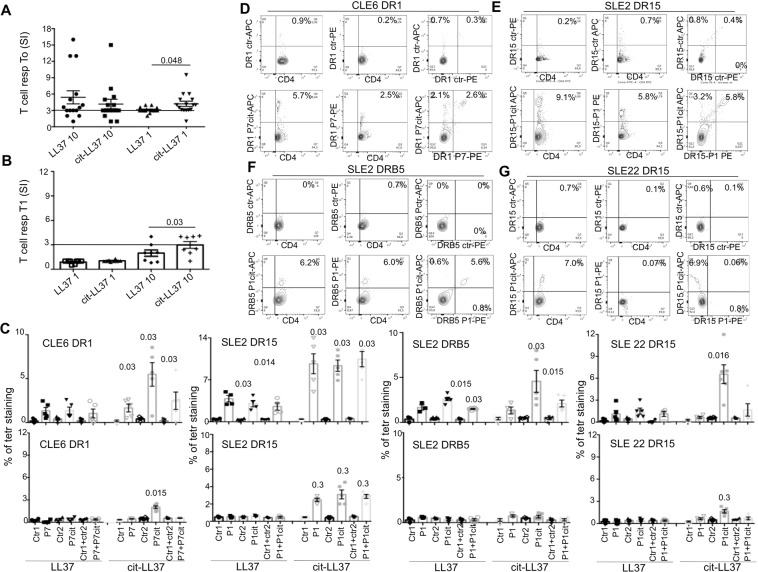
Cit-LL37 stimulates SLE/CLE LL37-specific T-cells better than native LL37. (**A**) T-cell proliferation (as in Fig. 1) of SLE LL37-specific T-cells to a low (1 μg/mL) and a high (10 μg/mL) dose of native LL37/cit-LL37 at T0 (**A**,**B**) in SLE patients at remission (T1, after therapy). Horizontal bars in A and B are the means, vertical bars are standard error of the mean, P values by Wilcoxon’s test. **(C)** Cumulative data of staining (as in Fig. S6A), with cognate/control tetramers (Table S5), showing the percent of CD4 T-cells double/single-stained with cognate peptide-MHC-tetramers (loaded with native LL37/cit-LL37 epitopes, and their respective controls), in PBMCs stimulated with native LL37, or with the same dose of cit-LL37, at T0 (upper panels), or at T1 (lower panels). Results are the mean plus/minus standard error of the mean of 5-to-6 experiments, performed with each patient. P-values were calculated between cognate tetramer and the correspondent control tetramer staining, by Wilcoxon signed-rank test. (**D–G**) Representative dual peptide-MHC-tetramer staining of four performed with each cognate peptide-MHC-tetramer and relative control (indicated), on SLE2/SLE22/CLE6 PBMCs stimulated for 7-days with cit-LL37 (peptide dose: 0.1 μg/mL for CLE6, 1 μg/mL for SLE2, 2 μg/mL for SLE22).

The identification of T-cells that promiscuously recognize the native and citrullinated forms of LL37 provided the tools to assess whether citrullination conferred advantages for T-cell activation. We used native or citrullinated peptides for which HLA binding capacity were similar (Fig. S7) (see also Methods). Proliferative responses to native-LL37 and cit-LL37 were similar in magnitude (Fig. 6A) when we used both antigens in high dose. However, T-cells responded better to cit-LL37 than the native counterpart at lower antigen doses. This happened in 12 LL37/cit-LL37 responders, while in three LL37-non-responders we observed no proliferation (Fig. 6A). Consistent with these findings, proliferation of PBMCs was still significant only in response to cit-LL37 (although at the highest dose) when patients were in remission (Fig. 6B). To substantiate these findings, we used peptide-MHC-tetramer staining to visualize the specificity, frequency and cross-reactivity of circulating SLE/CLE native LL37/cit-LL37-specific T-cells after a seven-day culture (gating strategy in Fig. S8A). In three patients analyzed, the frequency of peptide-MHC-tetramer positive T-cells was higher upon culture of PBMCs with cit-LL37 compared to native-LL37 (Fig. 6C, upper panels). This suggests that T-cells may have undergone preferential proliferative expansion when stimulated with cit-LL37. In the same culture conditions, PBMCs of HLA-matched HD did not stain significantly with LL37/cit-LL37 peptide-MHC-tetramers (Fig. S8B,C). Of interest, we observed heterogeneous responses among different patients (Fig. 6D–G). For instance, SLE2 and CLE6 had T-cells recognizing both native LL37 and cit-LL37, with a proportion of these cells exclusively recognizing cit-LL37 epitopes, while SLE22 had T-cells recognizing only cit-LL37. Furthermore, at remission, we observed not staining for native-LL37 in T-cells from SLE2, SLE22 and CLE6 PBMCs, while we still observed some staining upon stimulation with cit-LL37 (Fig. 6G, lower panel). Altogether, these data support the contention that cit-LL37, at least when presented in the context of selected HLA alleles, can act as stronger TCR-agonist for SLE T-cells, compared to native LL37. Responder T-cells remain detectable during clinical remission. Thus, these results suggest that the presence, at flares, of cit-LL37 in lupus target organs may enhance T-cell activation and would favor further damage and increased autoreactivity.

## Discussion

In this study, we report that T-cells belonging to the T_FH_-like/Th17 subsets recognize the autoantigen LL37 in SLE and are likely to favor the emergence of autoantibodies to LL37 and DNA/RNA in *in vitro* cultures and possibly *in vivo*. Consistent with this finding, we observed, by confocal microscopy, that LL37, DNA and IgG co-localize in SLE target organs, while statistical studies indicate that the magnitude of anti-LL37 antibody and T-cell reactivity correlate with each other and with SLE disease activity, as captured by SLEDAI, and decrease in patients with inactive disease. We provide evidence that the post-translational modification by citrullination of LL37 is readily detectable in SLE affected target organs (both skin and kidneys) and that, compared to native-LL37, cit-LL37 may further enhance T-cell responses. Altogether, these data strongly support the contention that adaptive immune responses directed against the autoantigen LL37 may participate to events relevant to SLE pathogenesis. Moreover, T/B-cell responses to LL37 are markers of active SLE. Indeed, by exploring distinct SLE cohorts, we found that anti-LL37 antibodies not only correlate with disease activity (SLEDAI) but also with circulating type I IFN serum levels. These data confirm and extend our previous report^9^, in contrast to observations by others that did not find a correlation between anti-LL37 antibodies and disease activity^27^. We ascribe this discrepancy to the larger proportion of untreated patients with severe disease and nephritis, coupled to a short disease duration, in our cohorts.

By using high-resolution confocal microscopy, we observed the presence of DNA filaments decorated with LL37 in kidney biopsies from patients with lupus nephritis, highly suggestive of LL37-DNA complexes formation *in vivo*. In the kidney, we could also demonstrate the co-localization of LL37 with IgG, suggesting the formation/deposition of LL37/DNA-immune complexes *in vivo*, where they might act by inhibiting NET-like structures degradation (38). The concomitant presence of Mx1, an IFN-α induced protein^23^ co-localizing with LL37 and IgG, provides supplemental evidence for a role of LL37-DNA complexes and anti-LL37 antibodies in IFN-I production *in vivo*^9,38^. Of note, anti-LL37 antibodies likely aggravate tissue inflammation by enhancing the uptake of LL37-DNA complexes into pDCs, an ability demonstrated *in vitro*^9^. The role and mechanism of NET formation in SLE is currently under debate^6,7,39–41^. Experimental models of SLE, although with several limitations, tend to exclude a pathogenic role for NETosis in SLE^42,43^, apparently in accordance with the finding that NETosis-defective individuals (with impaired NADPH activity) are more prone to lupus-like disease than HD^39–43^. On the other hand, NET-like structures form in both a NADPH-dependent, or independent manner, and NET-like cell death due to autoantibodies does not seem to rely on NADPH activity^40,44–47^. In our experiments, we observe that patient-derived anti-LL37 antibodies generated *in vitro* stimulate neutrophil extrusion of DNA filaments decorated by LL37. Thus, while we did not clarify in detail the mechanism that triggers the LL37-DNA complexes release, it is out of doubt that autoantibodies are actively responsible for NET-like structures formation *in vitro*. Of interest, the presence of consistent amounts of cit-LL37 in both skin and kidney, demonstrated for the first time in SLE tissues, makes also plausible the occurrence of the “leukotoxic hypercitrullination” (LTH) described by Andrade’s group in RA^25,26^. In LTH, neutrophils undergo a NET-like cell death upon membrane attack complexes (MAC) formation. Clear documentation of complement activation, consumption and deposition in tissues exists for SLE^48–50^. Thus, it is likely that LTH could explain the observed co-localization of C9 and cit-LL37 in SLE-affected kidneys. These data are anyway consistent with work showing that citrullination occurs in lupus kidneys (as consequence of inducible nitric oxide synthase –iNOS- activation^51^).

The main evidence that LL37 is a T-cell autoantigen in SLE reinforces the role of LL37 and anti-LL37 antibodies in SLE pathogenesis^9,13^. Interestingly though, T-cells also recognize LL37 as an autoantigen in psoriasis^17^, but autoantibodies to LL37 are infrequent in psoriasis^35^. In this respect, it is interesting that in both psoriasis and SLE the LL37-specific Th17-cells do exist and express Ror-ɣt but only in SLE, and not in psoriasis, they can express the T_FH-_markers Bcl-6 and high IL-21, at polyclonal and clonal levels. In addition, despite LL37 is over-expressed in RA synovia, UC gut and SSc skin, the magnitude of autoreactivity to LL37 in RA, UC and SSc is lower than in SLE^17,27,29–34^. Not only are T-cells specific for LL37 (and cit-LL37) more frequent in SLE than in RA and SSc, but correlation studies strongly suggest that these cells are instrumental for production of high affinity anti-LL37 antibodies exclusively in SLE, and functional experiments show that they do not help autoantibody production in psoriasis. The reasons why LL37 is a more potent target of both antibodies and T-cells in SLE than in RA and SSc, and why T_FH_-like cells are more easily apparent in SLE than in psoriasis are unclear. It is possible that LL37/LL37-DNA, and likely LL37-RNA-complexes^11^ reached the highest levels in SLE target organs most likely due to defective removal, rather than to enhanced release, which favors higher anti-LL37 immunity. Indeed, SLE has a recognized association with insufficient clearance of dead cells, and impaired nuclease activity^38,44,52–54^. In an old study, lower serum DNase1 levels associated with active SLE, the lowest DNAse I activity detected in patients with active renal involvement^52^. Persistence of LL37-DNA complexes can chronically activate DNA-binding receptors, and further fuel inflammation with production of the main actor in SLE, IFN-I. A non-mutually exclusive possibility is that the site where LL37 release and citrullination occur is determinant (the kidney). An argument to support this it is the high percentage of patients with severe SLE, accompanied by renal manifestations, who show high T-cell reactivity in our study. Indeed, impairment of NET-like structures degradation can correlate with lupus nephritis^38^. However, the magnitude of T-cell response to native/cit-LL37 also correlated with malar rash, suggesting skin involvement to be important for response to LL37.

Our study finds that T-cell responses to LL37 correlate with disease activity and anti-LL37-antibodies irrespective of whether T-cells are specific for native LL37 or cit-LL37. This raises the question as to whether LL37 citrullination is or is not involved in loss of T-cell tolerance in SLE. Since SLE T-cells can often cross-recognize native LL37/cit-LL37, citrullination may appear as a null event in the priming/activation of LL37 specific T-cells. Notably, though, in our experiments relatively low doses of cit-LL37 (more efficiently than native LL37), induced T-cell proliferation (as seen in BrdU-primary proliferation assays) and T-cell expansion, (as evidenced by increased percentage of peptide-MHC-tetramer staining). In addition, cit-LL37, more effectively than native LL37, recalled the residual LL37-specific T-cell responses persisting at low frequency at remission. Furthermore, in several patients, T-cells appeared exclusively activated by cit-LL37 and these T-cells helped anyway the production of antibodies to native LL37 *in vitro*. This is reminiscent of an *in vivo* animal model of autoimmunity described by Mamula *et al*., where a modified version of a self-antigen stimulated T-helper cells which drove antibodies against the original unmodified self-antigen^55^. Thus, it is possible that SLE LL37-specific T-cells, at least in a SLE sub-population, are primarily specific for cit-LL37, as cit-LL37 acts as a stronger agonist for these cells. Notably, recent works have linked a specific SLE-associated polymorphism (A20) with enhanced protein citrullination^56^, PAD4 polymorphisms with renal involvement^57^, and SLE-specific citrullinated antigens as antibody targets^58^, supporting a role for this post-translational modification in SLE pathogenesis. Cit-LL37-specific T-cells may thus cross-recognize native LL37 at a given threshold concentration, which may reach high levels during neutrophil activation. Our results could support this, since functional experiments and peptide-MHC-binding assays show that citrullinated LL37 epitopes have sufficient likelihood to be presented to T-cells by a wide array of MHC-II alleles, including DR15, DR1, DRB5, DR10, DR4, some of which (DR15, DRB5) are over-represented in SLE^59,60^.

In conclusion, both native LL37 and cit-LL37 are likely players in SLE, the former mainly for its adjuvant activity^9,10^, the latter for its increased T-cell immunogenicity. Less clear, and worth of further investigation, is the contribution of cit-LL37 to the immunogenicity of LL37 in psoriasis, a disease characterized by decreased citrullinated proteins in hyperproliferative epidermis, but also by neutrophil infiltrate^61^. Also, we are aware that the role of citrullination in autoreactivity in SLE could be restricted to the LL37-directed response, as additional post-translational modifications are reasonably considered dominant in SLE^62^. Of interest for the rheumatology field, the correlation between T-cell responses and antibody levels, and of both responses with disease activity (SLEDAI), renders LL37-directed responses interesting biomarkers, at least in patients that respond to LL37. It remain to systematically address whether a native/cit-LL37-directed autoreactivity marks nephritis, or skin rash, or both, and whether targeting of the dual effects of LL37 in the activation of both innate and adaptive immune responses^9–11,17^ is a novel avenue for therapy in SLE (as in psoriasis). For instance, we are currently investigating whether polyanionic compounds or LL37-specific aptamers reduce the immunogenicity of LL37, in addition to the concomitant block of its adjuvant activity in psoriasis^9^.

## Methods

### Study design

SLE/CLE blood (20 mL), kidney and skin biopsies, and blood from SLE, SSc, RA and UC patients were obtained in Rome, Italy, Policlinico Umberto I, Department of Internal Medicine and Medical Specialties - Rheumatology Unit, from University Hospital (CHUV), Department of Dermatology and Immunology, Lausanne, from the Swiss SLE cohort study (SSCS) and from Sandro Pertini Hospital, Rome. Sera and PBMCs from PSO patients were from University of Tor Vergata, Rome and Humanitas Hospital, Milan, Italy. Sera and skin from HD, matched for age and sex with SLE/CLE patients, were from blood Centers, Policlinico Umberto I, Italy and Geneva University Hospital, Switzerland. Disease activity in SLE patients was assessed by SLEDAI 2000^63^. For cutaneous lupus (CLE), we used CLASI^64^. For psoriasis (PSO) patients we used PASI (17). RA were diagnosed according to 2010 ACR/EULAR classification criteria^65^. UC disease activity was assessed by endoscopic Mayo 58 and UC patients with clinical and endoscopic activity (Mayo Score Full ≥3) were evaluated. Blood samples were collected at time of endoscopy^66^. SSc patients satisfied the ACR/EULAR 2013 classification criteria^67^. When possible, SLE/CLE patients did not take medications in the last three months before first blood sampling. Alternatively, we enrolled SLE/CLE patients during washout, and before entering a new protocol treatment. To corroborate data of the discovery cohort in different conditions, the replication cohorts (See Table S1) also included patients that were treated (exclusion criteria: patients treated with biologics). For longitudinal studies, the available patients who gave informed consent, were re-tested after taking medications, usually hydroxychloroquine and/or prednisone (see Table S1). Observation time between 6-to-36 months from first sampling (during a stable remission, after reaching an inactive disease state, SLEDAI < 4). HLA-Class II typing (HLA-DR) was performed in eight of the twenty-one SLE/CLE patients tested, in the reference center for HLA typing: Geneva University Hospital, Switzerland, and “Centro Transfusionale”, Rome, Italy. The assessed HLA-haplotypes are:

SLE2: DRB1*1501, DRB1*1601; CLE6: DRB1*0101, DRB1*1601; SLE1: DRB1*0301, DRB1*0401; SLE4: DRB1*0401, DRB1*1101; SLE17: DRB1*1101, DRB1 16*01. SLE22: DRB1*0301, DRB1*1501; SLE5: DRB1*1001, nd; SLE8: DRB1*0401, DRB1*0701.

All samples were obtained upon approval by Ethic Committees of University La Sapienza, Tor Vergata, Sandro Pertini Hospital in Rome, and Humanitas Hospital, Milan, Italy, and of CHUV and Swiss Ethics, Switzerland. All blood and tissue donors gave informed consent, according to Helsinki declaration.

### Antigens

We list the antigenic peptides used in this study in Table S3. LL37 and HBD3 were from Innovagen. LL37 was also from Proteogenix (France). REV and scramble LL37 (SCR) peptides from AnaSpec Inc. Overlapping LL37-peptides (Supplementary Table 1^17^), as well as the citrullinated LL37 (citrullinated at all five arginin positions) were made by Anawa, Trading SA (Zurich, CH) or Citomatik (Italy). Byotinilated native LL37 and cit-LL37 were also from Anawa. HNP_1–3_ was from Hycult Biotechnology. Citrullinated SCR was synthesized by Biomatik (Italy). Citrullinated and non-citrullinated vimentin and enolase were synthesized by Genscript as described^68^. Citrullinated and non-citrullinated vinculin and HA peptides synthesized by Fmoc as described^69^.

### Generation of anti-LL37/cit-LL37 antibodies

Antibodies Mab137 and Mab142, specific for native LL37 (Mab137) and cit-LL37 (Mab142) were produced in the Antibody Facility of University of Geneva, CMU, CH (http://www.unige.ch/antibodies) as described^70^. For confocal detection a secondary anti-mouse antibody conjugated with AlexaFluor-647 or AlexaFluor-488 was used (Abcam). Isotype controls were from Hycult.

### Isolation of blood pDCs

Buffy coats of HD were from Centro Trasfusionale, Policlinico Umberto I, Rome, IT. After separation of mononuclear cells by Ficoll centrifugation, pDCs were purified as described^9,10^, by using the Diamond Plasmacytoid Dendritic Cell Isolation Kit (Miltenyi Biotec) to obtain 99% purity.

### ELISA for autoantibody detection in sera and culture supernatants

Anti-native/cit-LL37, other antigen antibodies (anti-DNA and anti-RNA), and total anti-human IgG were measured by ELISA as described^9^. Briefly, 96-well flat-bottom plates (Non-Binding surface polystyrene, Corning, USA) are coated with 2 μg/mL of native LL37/cit-LL37 or control proteins or human DNA or human RNA^9^ or anti-human IgG antibody (Sigma) in carbonate buffer (0.1 M NaHCHO_3_, pH 9) for 2 hours (or over night) and washed four times with PBS 0.1% Tween-20. This washing buffer was used for washing at all steps. The blocking buffer containing 2% bovine serum albumin (BSA, Sigma) in PBS was used for at least 1 hour (or over night) to saturate unspecific binding sites. After washing, sera were diluted at various concentrations (usually 1:100 or 1:200) in PBS 2% BSA (cell culture supernatants were diluted 1:2, 1:4 or 1:10) followed by 1 hour incubation with a horseradish peroxidase–conjugated (HRP) goat anti-human IgG (Sigma-Aldrich), diluted 1:10,000 in PBS. The color was developed for 5 minutes with 3,3’,5,5’-tetramethylbenzidine (TMB) substrate (Sigma-Aldrich). The reaction was stopped by adding 50 μl of 2 N H_2_SO_4_, and absorbance determined at 450 nm with a reference wavelength of 540 nm. Anti-dsDNA in sera of lupus patients were detected by the standard clinical test Kallestad *Crithidia luciliae* Complete Kits for detection and semi-quantitation of autoantibodies to native DNA (nDNA) antigens by indirect fluorescence antibody (IFA) procedure. ELISA for detection of anti-tetanus antibodies (anti-TT) was from Abnova (Taiwan).

### Purification of neutrophils, NET-induction and quantification

Neutrophils were isolated from peripheral blood of SLE patients with Polymorphoprep (Euroclone). Yield, measured by flow cytometry for CD15 expression, was 95 to 97% (the remaining cells were eosinophils and monocytes expressing low levels of CD15)^9^. Purified neutrophils were seeded at 5 × 10^5^ cells in 200 μL of complete medium and stimulated with anti-LL37 (clone 8A8.1, IgG2b, 10 μg/mL) as in our previous study^9^, or with IgG control antibodies or LL37-positive and LL37-negative supernatants (diluted 1:4 in medium). The release of NET-DNA was measured in cell-free supernatant collected after 3 hours of activation following addition of the PicoGreen using the PicoGreen assay kit. Samples were excited at 480 nm and read at 530 nm with a fluorimeter.

### Production of recombinant HLA class II molecules

Human recombinant HLADRA1*01:01, HLA-DRB1*01:01, and HLA-DRB1*15:01 were expressed in *E. coli* and purified in a series of liquid chromatography based steps^71^.

### Peptide-MHC class II binding assays

We performed peptide-MHC binding assays by two different protocols by ImmunAware, Denmark and Benaroya Institute (USA). In the first protocol, binding was measured using a Luminescent Oxygen Channelling Immunoassay (LOCI), marketed by Perkin Elmer as AlphaScreen^72^. This is a nonradioactive bead-based homogenous proximity assay, where the donor beads containing a photosensitizer compound upon excitation with light at a 680 nm wavelength, converts ambient oxygen to energy-rich, short-lived singlet oxygen; that is transferred to a acceptor beads in close proximity only. The acceptor beads can respond to singlet oxygen with a luminescence/ fluorescence cascade leading to an amplified signal in the 520–620 nm range. As one tag binding to the donor beads: a biotin group engineered unto the HLA β chain. As the other tag binding to the acceptor beads: conformation-dependent HLA class II specific antibody (L243). Thus, only when a fully folded complex is present will the donor and acceptor beads come in sufficient close proximity and give a measurable signal. Biotinylated recombinant HLA class II molecules were diluted from an 8 M urea buffer into a folding buffer (PBS, 20% glycerol, Protease inhibitor mix) with peptide concentrations ranging from 0.13 nM to 10,000 nM. The analysed peptides were tested in 5-to-8 five-fold concentration titrations spanning from 20 μM to 0.3 nM. The reaction mixtures were incubated for 48 h at 18 °C to allow for peptide-HLA complex formation reaching steady state. After incubation, 15 μL of the folding mixture was transferred to a 384-well Optiplate (PerkinElmer) followed by addition of 15 μL of a solution containing L243 acceptor beads and streptavidin donor beads (final concentration of beads was 5 μL/mL). The plates were incubated over night at 18 °C, and then measured in enVisionTM, Perkin Elmer. Assay signals measured in OD450 were were plotted against the offered peptide concentrations, and analyzed by non-linear regression using GraphPad Prism. The peptide concentration resulting in half saturation, the half maximal effective concentration (EC50), was estimated by fitting the experimental data to the equation: Y = Bmax*X/(KD + X), where Y is the OD450 measurement of complexes formed and X is the concentration of ligand (peptide) offered. The EC50 approximates the KD as long as the receptor concentration used is less than the KD thus avoiding ligand depletion.

For the second binding protocol^72,73^, we used purified recombinant DRB1*0401, DRB1*1601 or DRB5*0101 and DRB1*1001 proteins for *in vitro* peptide-binding assays. Increasing concentrations of each non-biotinylated test peptide was incubated in competition with a previously determined fixed concentration of biotinylated reference peptide (1 µM of NFIRMVISNPAAT for DRB1*0401 and DRB1*1001, 1.5 µM of NFIRMVISNPAAT for DRB1*1601, and 0.8 µM PKYVKQNTLKLAT for DRB5*0101) and captured in microtiter plate wells coated with 10 μg/mL of L243 (anti-HLA-DR) antibody. After washing, residual biotinylated reference peptide was labelled using europium conjugated streptavidin (Perkin Elmer) and quantified using a Victor3 D time resolved fluorimeter (Perkin Elmer). We also calculated the IC50 binding values from the resulting curves, as the peptide concentration needed for 50% inhibition of reference peptide binding.

## Supplementary information

Supplementary information.
