# Supplementary material for: Native/citrullinated LL37-specific T-cells help autoantibody production in Systemic Lupus Erythematosus
Source: Sci Rep. 2020 Apr 3;10:5851. doi: 10.1038/s41598-020-62480-3 (PMC7125190; doi:10.1038/s41598-020-62480-3)
Supplement: Supplementary file 1 — Supplementary information. [file 41598_2020_62480_MOESM1_ESM.pdf]

SUPPLEMENTARY INFORMATION

Native/citrullinated LL37-specific T-cells help autoantibody production in Systemic Lupus Erythematosus

R. Lande[1,2], R. Palazzo[1], N. Gestermann[2], C. Jandus[3], M. Falchi[4], F. Spadaro[5], V. Riccieri[6], E. A. James[7], A. Butera[1], M. Boirivant[1], L. Feldmayer[2], I. Surbek[2], J. Di Lucca[2], F. Stuber[2], F.R. Spinelli[6], E. Botti[8], B. Marinari[8], L. Bianchi[8], R. Pica[9], B. Cerbelli[10], K. Giannakakis[11], S.E. Auteri[1,6], I. Daniels[12], L. Durrant[12], S Horstman[7] A. Costanzo[13], P. Romero[3], C. Alessandri[6], F. Conti[6], G. Valesini[6], M. Gilliet[2], C. Chizzolini[14], and L. Frasca[1,2,14]*.

[1]Istituto Superiore di Sanità, National Centre for pre-clinical and clinical drug research and evaluation, Pharmacological research and experimental therapy Unit, 00166, Rome, Italy;
[2]University Hospital CHUV, Dept. of Dermatology, 1011 Lausanne, Switzerland;
[3]Translational tumor immunology group, Department of fundamental oncology, University of Lausanne, 1066 Epalinges, Switzerland.
[4]Istituto Superiore di Sanità, National AIDS Center, 00166, Rome, Italy.
[5]Istituto Superiore di Sanità, Confocal Microscopy Unit, Rome, Italy
[6]Division of Rheumatology, Internal medicine and medical specialties, University Sapienza, Rome, Italy;
[7]Translational Research Program, Benaroya Research Institute at Virginia Mason, Seattle, WA, USA;
[8] Dermatology Unit, Department of Systems Medicine University of Tor Vergata, Rome, Italy;
[9] "Sandro Pertini" Hospital, IBD, GE Unit, Rome, Italy;
[10] Department of Radiological, oncological and anatomo-pathological sciences, Sapienza University, Rome, Italy, current address: Department of Medico-Surgical Sciences and Biotechnologies, Sapienza University, Latina, Italy;
[11]Department of Pathology, Sapienza University Rome,Italy;

[12]Academic Department of Clinical Oncology, University of Nottingham, Nottingham, UK;
[13] Skin pathology laboratory, Dermatology Unit, IRCCS Humanitas, Milano, Italy;
[14] Immunology and Allergy, Pathology and Immunology, University Hospital and Medical School, Geneva, Switzerland.

Supplementary Figures and legends to Figures

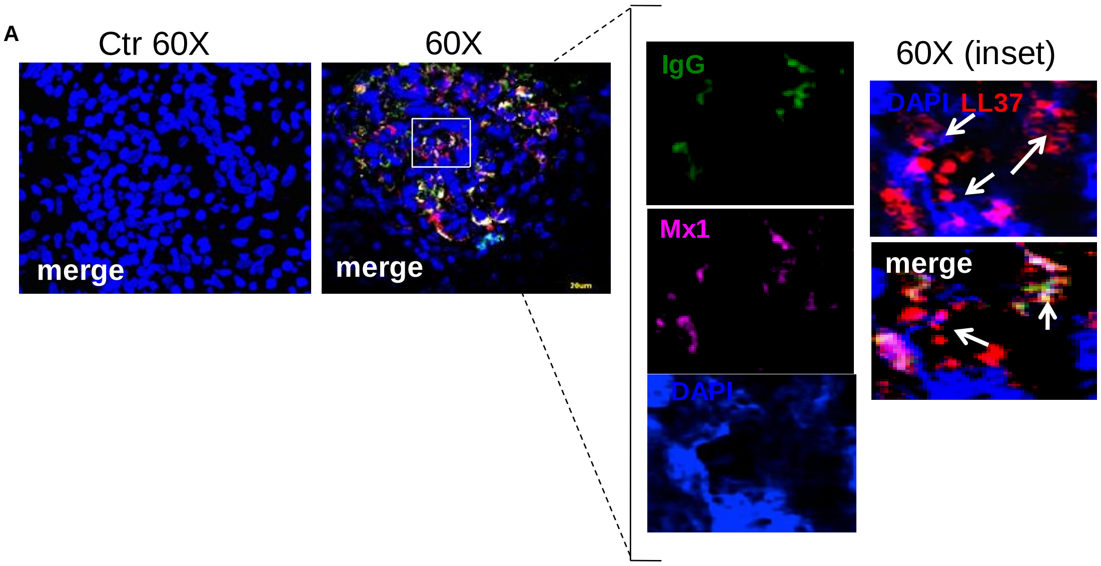

Figure S1. IgG/Mx1 expression in the vicinity of LL37-DNA complexes in SLE-affected kidney. **(A)** Magnification of staining in Fig. 1G to show DNA filaments decorated by LL37 in proximity of MxA and IgG staining, control antibody staining is also shown (ctr merge).

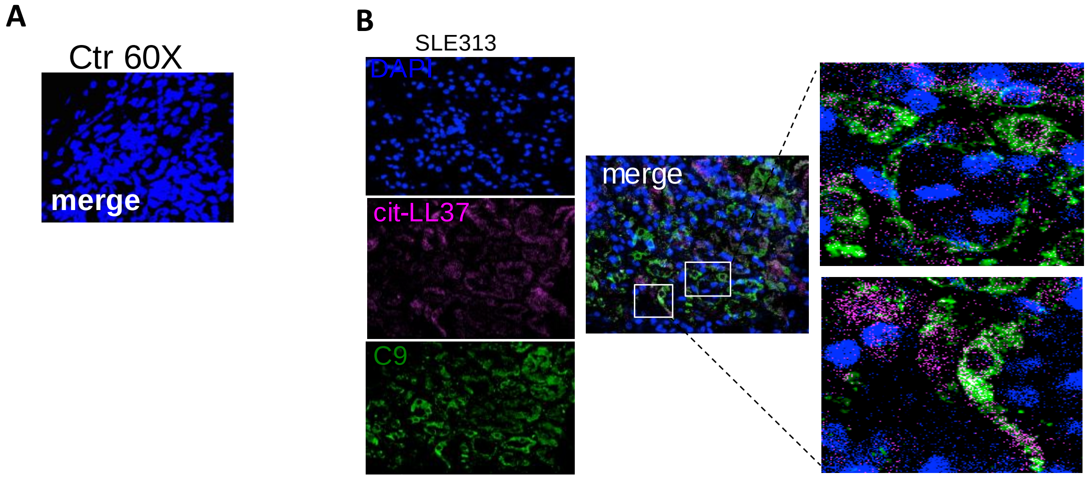

Figure S2. Citrullinated LL37 (cit-LL37) and C9 complement are both present and co-localize in SLE affected kidney. (A,B) Staining of SLE-affected kidney for cit-LL37 and complement C9 as indicated. Control antibody staining in (A). Representative staining of one patients out of staining from a total of three SLE patients renal biopsies.

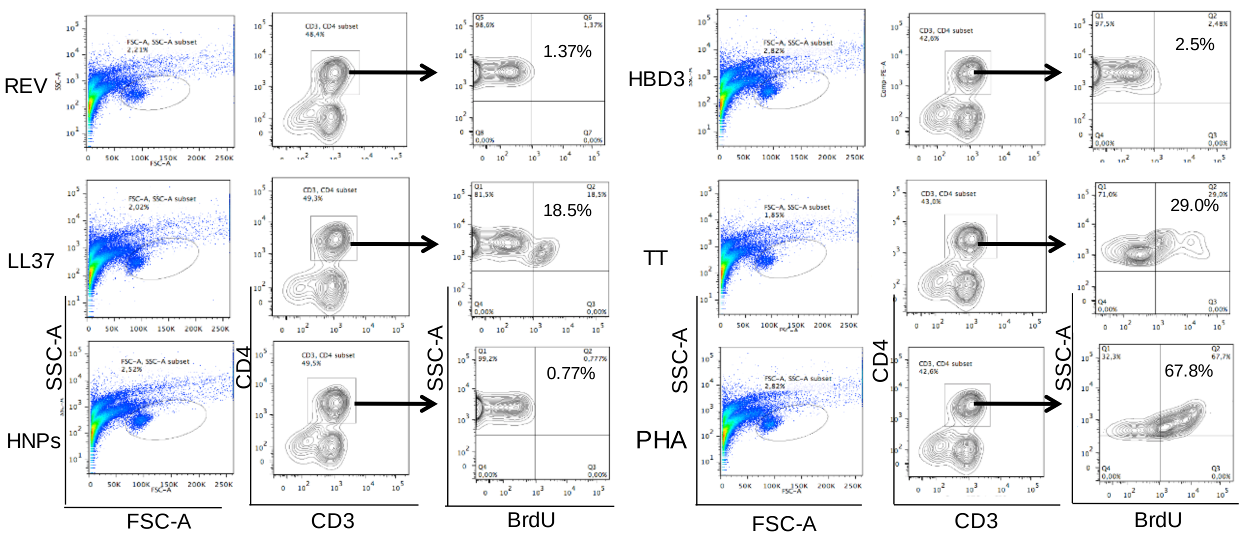

Figure S3. SLE T-cells proliferate to LL37. Gating strategy for measuring BrdU incorporation by CD3+CD4+ cells of one SLE patient, stimulated for 6 days with: control REV peptide (10µg/mL), LL37 (10µg/mL), HNPs (10µg/mL), HBD3 (5µg/mL), TT antigen (10µg/mL) or PHA (2µg/mL). Percentage of BrDU+CD3+CD4+ cells was evaluated by flow cytometry and is given in the representative contour plots.

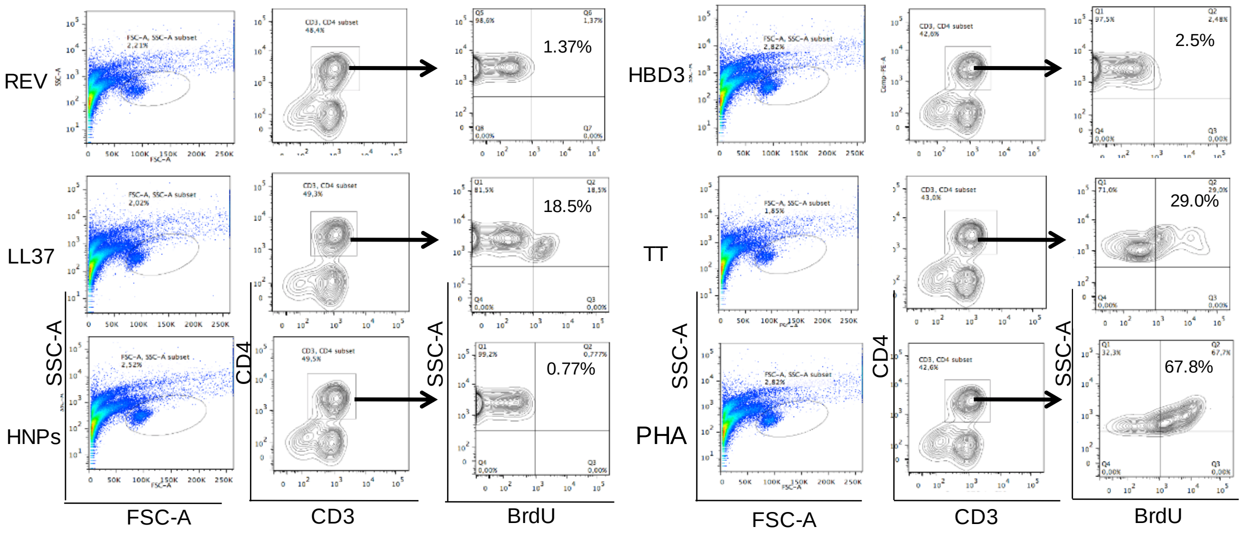

Figure S3. SLE T-cells proliferate to LL37. Gating strategy for measuring BrdU incorporation by CD3+CD4+ cells of one SLE patient, stimulated for 6 days with: control REV peptide (10µg/mL), LL37 (10µg/mL), HNPs (10µg/mL), HBD3 (5µg/mL), TT antigen (10µg/mL) or PHA (2µg/mL). Percentage of BrDU+CD3+CD4+ cells was evaluated by flow cytometry and is given in the representative contour plots.

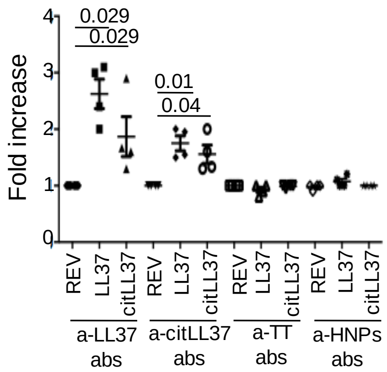

Figure S5. T-cell help for antibody production of LL37/cit-LL37-specific T-cells is restricted to LL37/cit-LL37 and it is not bystander. SLE PBMCs were stimulated with native-LL37 or cit-LL37 or control REV and anti-native-LL37/anti-cit-LL37 and anti-TT- or anti-HNP-antibody fold increase with respect to untreated cells, was assessed by ELISA. Horizontal bars are the means, vertical bars standard errors of the mean, P values by Wilcoxon's test.

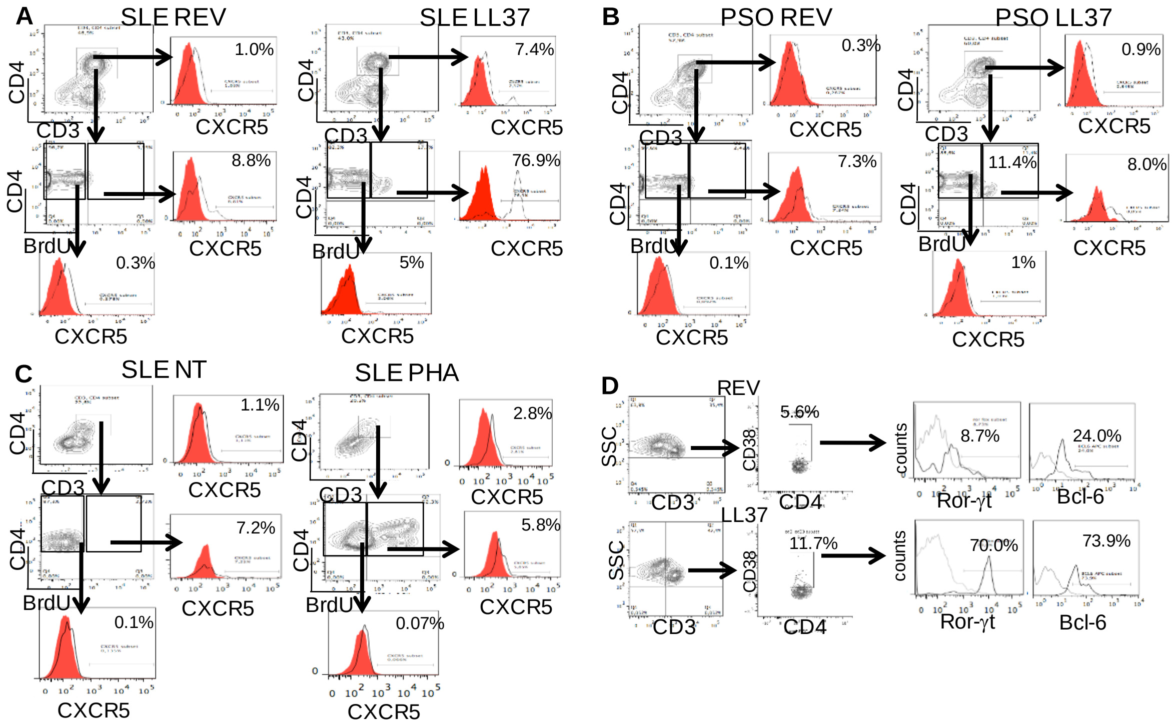

Figure S6. SLE CD4^{+} cells express markers of T$_{FH}$ /Th17-cells. (**A,B,C**) Flow cytometry gating strategy to measureexpression of CXCR5 by T-cells proliferating (Brdu+) to LL37 or control REV (**A,B**) peptides, or proliferating to polyclonal stimulation (PHA) **(C)**. **(D)** Gating strategy for assessment of Bcl-6 or Ror-γt in CD4+ T-cells cultured for 48 hours with REV or LL37 peptides (by intracellular staining). To make staining for Bcl-6 and Ror-γt apparent we gated on CD4 T-cells that showed the highest expression of CD38 **(D)**.

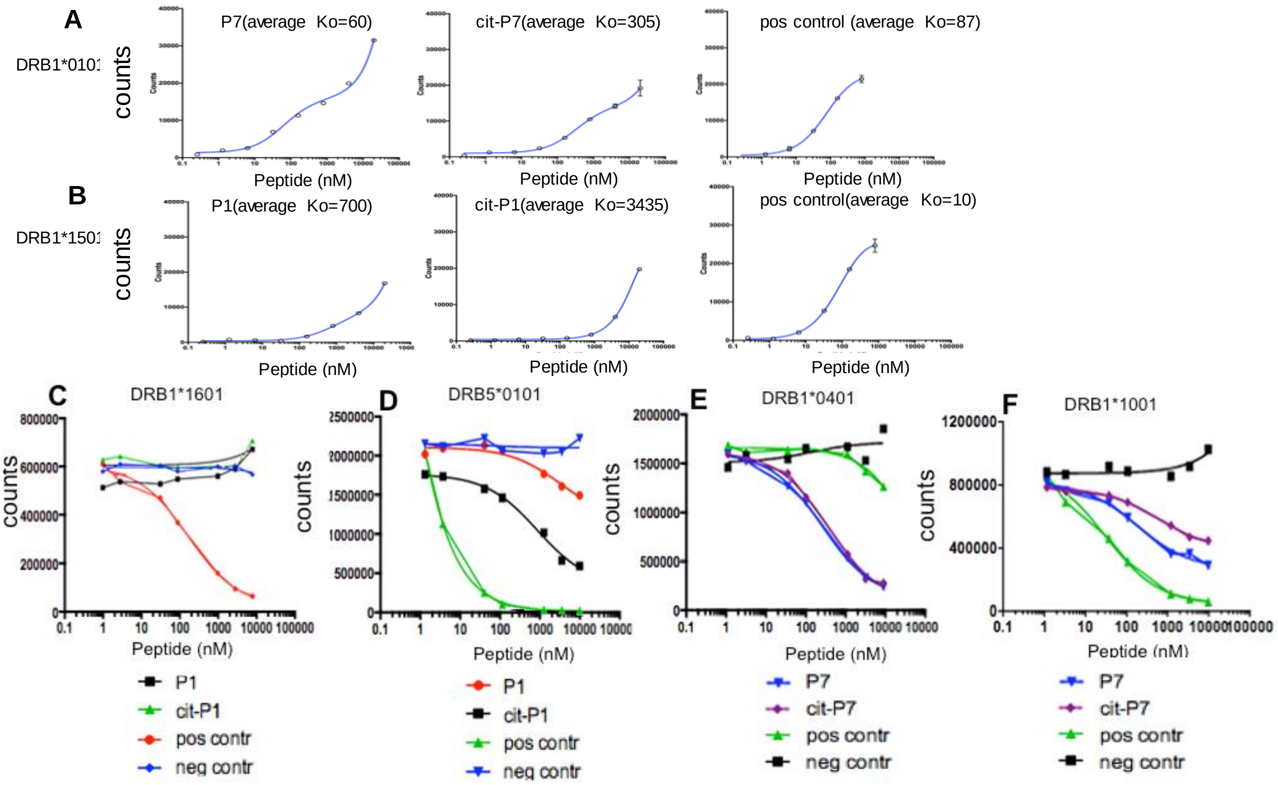

Figure S7. LL37 epitopes bind HLA-DR molecules in their native and citrullinated form. **(A,B)** Binding of LL37-peptides to HLA-DR1 (DRB1*0101) and to HLA-DR15 (DRB1*1501) was evaluated by a HLA-DR folding assays and detected by Luminescent Oxygen Channelling Immunoassay (LOCI) described in MM. Reported one representative experiment of two performed. **(C-F)** Binding to DR16 (DRB1*1601), DRB5 (HLA-DRB5*0101), DR4 (HLA-DRB1*0401) and DR10 (DRB1*1001) was evaluated by peptide competition assay as in MM, and evaluated fluorimetrically. Reported one representative experiment of two performed.

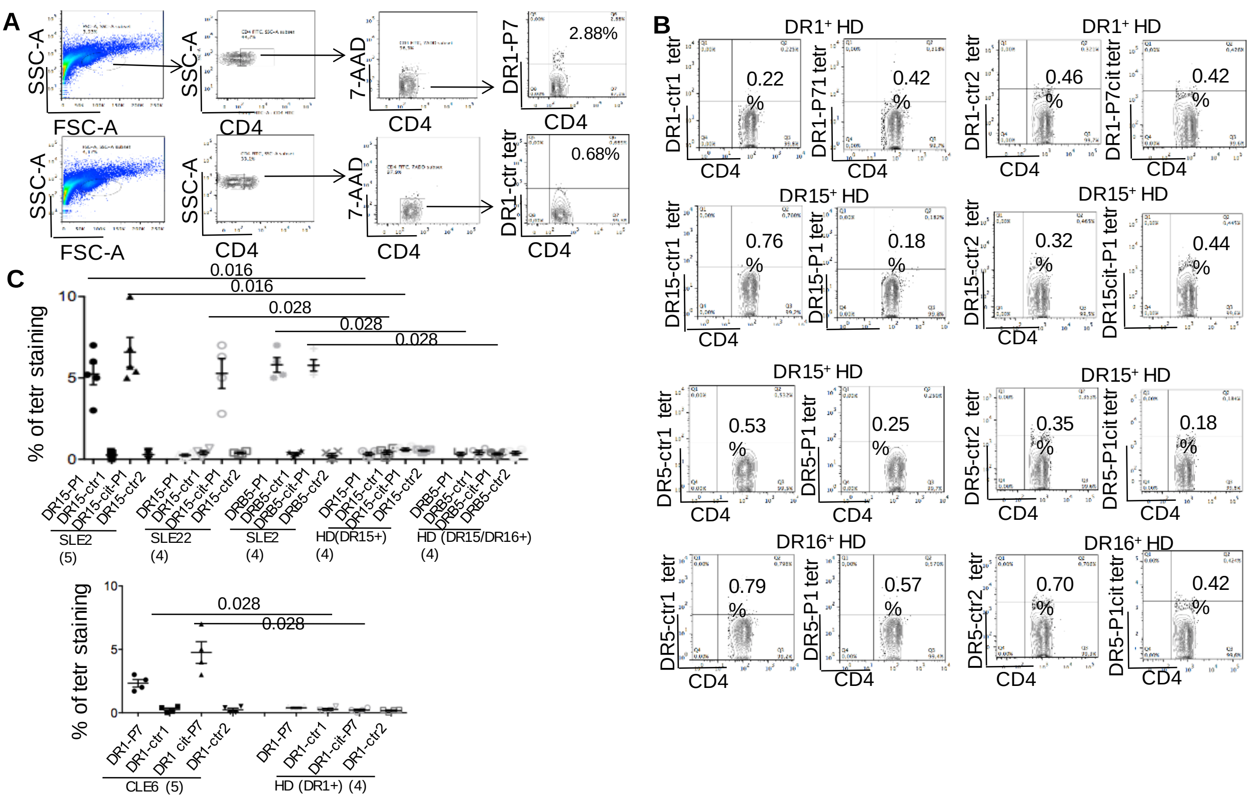

Figure S8. Peptide-MHC-tetramers stain SLE/CLE and not HD T-cells.

(A) Gating strategy for tetramer staining with cognate (DR1-P7 tetr) or control tetramer in PBMC of CLE6 stimulated for 7 days with LL37. Gate on CD4+ alive cells (7AAD-CD4+ cells). Represented one experiment of 6 performed with CLE6 PBMCs. **(B)** HLA-DR matched HD PBMCs, cultured with LL37 or cit-LL37 (5 mg/mL) for seven days were stained with cognate/control peptide-MHC-tetramers (Table S4). One representative set of flow cytometry contour plots of 4 independent experiments performed in 12 different HD for each peptide-MHC-tetramer pair (cognate tetramers and control tetramers). **(C)** Cumulative data of B, shown are results of repetitive stainings of SLE2, SLE22 and Cle6 for the same tetramer pairs. Horizontal bars are the mean, vertical bars are SEM, P values by Mann-Whitney test (two tails).

Table S1: Clinical characteristics of the studied cohorts

SLE cohorts	Discovery (40)	Replication1 (39)	Replication2 (22)
Age, mean (range) years	39 (30–75)	45 (32–60)	47 (25–75)
Sex (M/F)	6/34	7/32	7/15
Disease duration, mean (range) months	24 (6–60)	48 (15–60)	35 (16-71)
SLEDAI	9 (0-24)	7 (0-31)	7 (0-22)
Active disease (SLEDAI>4)	23/40 (57%)	26/39 (66%)	12/22 (54%)
Severe SLE (SLEDAI>10)	17/40 (42%)	5/39 (13%)	6/22 (27%)
Prednisolone use (%)	40%	50%	50%
Rash	31/40 (77%)	11/39 (28%)	5/22 (27%)
Renal involvement	15/38 (39%)	5/39 (13%)	10/22 (45%)
CNS involvement	0/40 (0%)	1/39 (0.2%)	0/22 (0%)
Arthritis	5/38 (13%)	30/39 (77%)	4/22 (12%)
ANA	34/40 (85%)	30/39 (77%)	16/22 (73%)
Anti-dsDNA	24/40 (60%)	25/39 (64%)	16/22 (73%)

Controls	SSc (40)	PSO (35)	RA (70)	UC (58)	HD (50)
Age, mean (range) years	59 (30–75)	45 (25–70)	50 (26–60)	48(20-76)	45(28-60)
Sex (M/F)	4/26	25/20	25/45	35/23	29/21
Disease duration, mean (range) months	60 (1–100)	48 (24–120)	181.5 (60-348)	144 (0-432)	N/A
PASI	N/A	7.5 (2-25)	N/A	N/A	N/A
DAS28	N/A	N/A	4 (1.8-5.8)	N/A	N/A

CNS, Central Nervous System; dsDNA, anti-DNA double strand; N/A, not applicable

Patient	CLASI*	Subtype *
CLE5	2	DLE
CLE6	24	SCLE
CLE3	0	DLE
CLE11	0	DLE
CLE8	1	DLE

Table S2 Studied CLE patients. Disease activity (CLASI) and disease subtypes of CLE patients analyzed (DLE, discoid lupus erythematosus, SCLE, subacute CLE).
*Classification and disease activity at time of the first test (BrdU-incorporation, serum antibody reactivity, tetramer staining).

Table S3. Peptides used in the study:

Name			aa lengh
P1	MIX1	LLGDFFRKSKEKIGK	(15)
P2	MIX1	FFRKSKEKIGKEFKR	(15)
P3	MIX1	SKEKIGKEFKRIVQR	(15)
P4	MIX1	IGKEFKRIVQRIKDF	(15)
P5	MIX2	FKRIVQRIKDFLRNL	(15)
P6	MIX2	VQRIKDFLRNLVPRT	(15)
P7	MIX2	KDFLRNLVPRTES	(13
cit-P1		LLGDFFR(cit)KSKEKIGK	(15)
cit-P7		KDFLR(cit)NLVPR(cit)TES	(13)
LL37		LLGDFFRKSKEKIGKEFKRIVQRIKDFLRNLVPRTES	(37)
cit-LL37		LLGDFFR(cit)KSKEKIGKEFKR(cit)IVQR(cit)IKDFLR(cit)NLVPR(cit)TES	(37)
REV		SETRPVLNRLFDKIRQVIRKEFEKGIKEKSKRFFDGLL	(37)
cit-REV		SETR(cit)PVLNR(cit)LFDKIR(cit)QVIR(cit)KEFEKGIKEKSKR(cit)FFDGLL	(37)
SCR		GLKLRFEFSKIKGEFLKTPEVRFRDIKLKDNRISVQR	(37)
cit-SCR		GLKLR(cit)FEFSKIKGEFLKTPEVR(cit)FR(cit)DIKLKDNR(cit)ISVQR(cit)	(37)
HBD3	HNPs$_s$	GIINTLQKYYCRVRGGRCAVLSCLPKEEQIGKCSTRGRKCCRRKK	(45)
HNP$_1$	HNPs$_s$	ACYCRIPACIAGERRYGTCIYQGRLWAFCC	(30)
HNP$_2$	HNPs$_s$	CYCRIPACIAGERRYGTCIYQGRLWAFCC	(29)
HNP$_3$	HNPs$_s$	DCYCRIPACIAGERRYGTCIYQGRLWAFCC	(30)
HA$_{306\text{-}320}$ (15)		CPKYVKQNTLKLATG	
cit-HA$_{306\text{-}320}$		CPKYVKcitNTLKLATG	(15)
Vinculin		LLAVAATAPPDAPNREEVFDERAANFE	(28)
Cit-vinculin		LLAVAATAPPDAPNREEVFDER(cit)AANFE	(28)
Vimentin28		RSYVTTSTRTYSLGSALRPSTS	(22)
cit-vimentin28		R(cit)SYVTTSTR(cit)TYSLGSALR(cit)PSTS	(22)
Enolase21		VIGMDVAASEFFRSGKYDLD	(20)
cit-enolase21		VIGMDVAASEFFR(cit)SGKYDLD	(20)

Table S4. Peptide-MHC-tetramers used in the study:

name	HLA-DR	epitope sequence	antigen	fluorochrome
DR15-P1 tetr	HLA-DRB1*1501	LLGDFFRKSKEKIGK	LL37	(PE)
DR15-P1cit tetr	HLA-DRB1*1501	LLGDFFR(cit)KSKEKIGK	Cit-LL37	(APC)
DR15-Ctr1 tetr	HLA-DRB1*0101	TNPLIRHENRMVLASTT	M1-169-185*	(PE)
DR15-Ctr2 tetr	HLA-DRB1*0101	PVSKMRMATPLLMQA	CLIP	(APC)
DRB5-P1tetr	HLA-DR5*0101	LLGDFFRKSKEKIGK	LL37	(PE)
DRB5-P1cit tetr	HLA-DRB5*0101	LLGDFFR(cit)KSKEKIG	Cit-LL37	(APC)
DRB5-P1Ctr1	HLA-DR5*0101	PVSKMRMATPLLMQA	CLIP	(PE)
DRB5-P1Ctr2	HLA-DRB5*0101	PVSKMRMATPLLMQA	CLIP	(APC)
DR1-P7 tetr	HLA-DRB1*0101	KDFLRNLVPRTES	LL37	(PE)
DR1-P7cit tetr	HLA-DRB1*0101	KDFLR(cit)NLVPR(cit)TES	Cit-LL37	(APC)
DR1-Ctr-1	HLA-DRB1*0101	PKYVKQNTLKLAT	HA**306-318	(PE)
DR1-Ctr-2	HLA-DRB1*0101	PVSKMRMATPLLMQA	CLIP	(APC)

List of the peptide antigens used in this study.
*Matrix protein-1 (Flu)
**Hemagglutin

SUPPLEMENTARY METHODS:

Antibodies and reagents for flow cytometry, confocal microscopy and immunohistochemistry

Antibodies to CD4, CD8, CD3, conjugated with various fluorochromes [FITC, phycoerythrin (PE), peridinin-chlorophyll-protein (PerCp), allophycocyanin (APC), PE-Cy7] were from BD Biosciences or eBiosciences (San Diego, CA). For further LL37-specific T-cell characterization, FITC-, APC-, PE-, PerCp-, PerCpCy5.5-, APC-Cy7-, AlexaFluor488-conjugated CCR6, -CLA, -CXCR5 (FAB190), PD1, -ICOS, -CD38, CD19, CD20, CD27, CD138 antibodies were purchased from BD Biosciences, eBiosciences, Novus Biologicals (Littleton, CO), R&D (Minneapolis, MN), Invitrogen Life Technologies. FITC or AlexaFluor 488, APC, PE, PerCp-conjugated anti-human-IFN-ɣ, -IL-17, -IL-22, -IL-21 were from BD Pharmingen, eBiosciences, BioLegend (San Diego, CA) or R&D Systems, as well as appropriate isotype-matched controls. PerCp-7-AAD was from BD Pharmingen, DAPI was from Invitrogen. APC-anti-human Bcl-6 (clone BCL-UP) and PE-anti-human Ror-ɣt (Clone AFKJS-80) and appropriate isotype control were from eBiosciences. Anti-CD15 was from BD Pharmingen and PicoGreen was from Invitrogen (PicoGreen assay kit). Rabbit anti-LL37 was from Innovagen (Sweden). Anti-LL37 for NET detection on neutrophils isolated from patients was biotinylated (Innovagen), and streptavidin AlexaFluor 488 from Thermo Fisher Scientific (USA) was used to reveal staining.

Stimulation of pDCs

Purified pDCs were seeded into 96-well round-bottom plates at 3.5/5 × 10^4 cells per well in 200 µl RPMI 1640 (GIBCO), supplemented with 15% fetal calf serum (FCS), 1% Hepes and 1% sodium piruvate. LL37, cit-LL37 and LL37-shorter peptides were premixed with total human genomic DNA (from Roche, CH or extracted from PBMC of HD) (10 µg/mL). After 30 minutes incubation at room temperature the mix was added to the pDC cultures. IFN-α in supernatants was measured by enzyme linked immunosorbent assay (ELISA) (MabTech, France) after 24 hours.

T-cells proliferation assay

PBMCs were purified, from EDTA-treated blood, on Ficoll-Hypaque (Pharmacia Fine Chemicals, Uppsala, Sweden) were incubated (10^5 cells/well) in 96-well-flat-bottom-microplates (BD) in T cell-medium (RPMI 1640, 10% heat-inactivated human serum, HS, Gibco), 2 mM L-glutamine, 10 U/mL penicillin, and 100 μg/mL streptomycin), with/without peptides. We performed assays on fresh cells, within 1-to-3 hours from collection, or on PBMCs frozen in 90% FCS-10%DMSO, whose viability we assessed by Trypan blue exclusion, at inverted microscope. Recovery of live cells was between 65 and 85 % of the frozen number. At day3 and 5, BrdU was added (1 μg/mL). BrdU-incorporation was detected by APC-labeled anti-BrdU antibody (BD Pharmingen), after surface staining by flow cytometry. Internal control for T-cell viability and proliferation was PHA treatment (2 μg/mL). SI for proliferation was calculated by dividing percent of BrdU-staining in the presence of each peptide used to stimulate cells by percent of BrdU-staining in the absence of peptide stimulation. SI for proliferation was considered positive when≥3. This cut-off was obtained by calculating the mean+2DS [1.5 + 2 (0.6)] (of the stimulation indexes calculated as above) of the proliferation of HD. Basal % of proliferation was set using, as reference, an isotype matched antibody staining (APC-isotype control and) and never exceed 5% of cell proliferation value (background proliferation). Assays were repeated twice with each patient. In rare cases in which background proliferation exceed antigen-specific proliferation the assay was repeated three times. Concomitant phenotype analysis of LL37-responder T-cells included staining for CD4, CD8, CXCR5, PD1, ICOS, CCR6. Ror-ɣt and Bcl-6 were measured by intracellular staining and flow cytometry, after 48 hours culture with LL37 or control antigen, and were analyzed on CD38^{high}CD4 T-cells.

Generation/characterization of T-cell lines/clones

PBMCs were stimulated ($2x10^6$ cells/mL) with LL37 for 7 days and re-stimulated (10 days) with LL37-pulsed (10 μg/mL) autologous-ɣ-irradiated-(30Gy)-PBMCs in T-cell medium in the presence of human/recombinant (hr)IL-2 (Boehringer-Mannheim, Indian, USA). T cell lines-specificity was analyzed by using peptide-pulsed (0.1-10 μg/mL) autologous-irradiated PBMCs (30Gy) or lymphoblastoid-cell-lines (B-LCLs) (150Gy, 33). BrdU was added at day 3 and 5; T-cells were

analyzed by flow-cytometry (after staining by BrdU-detection kit, BD Pharmingen). Before cloning, bulk-cultures were stimulated with autologous-irradiated PBMCs and peptide over night, and stained for CD3 and CD4 and with the "Cytokine secretion assays kits" (MACS, Miltenyi Biotec) to detect IL-2-, IFN-ɣ- and/or IL-17-secretion and CD38 as an activation marker, and sorted by a cell-sorter (MoFlo Astrios, Beckman Coulter). Dead cells were excluded by DAPI staining. Sorted activated $CD4^{+}CD3^{+}$ cells, secreting cytokines were cloned by limiting dilution in Terasaki plates (Nunc Microwell, Sigma-Aldrich) in the presence of allogeneic-irradiated (10^{4}) PBMCs activated by phytohaemagglutinin (1 µg/mL, PHA, Murex, 0.5 cells/well) and 100 U/mL of hrIL-2. Clones reactivity and HLA-restriction were analyzed using HLA-matched-homozigous B-LCLs, pulsed with peptides.

Measurement of cytokine production in primary T-cells and T-cell lines/clones

Cytokines secretion was detected by ELISA (Quantikine; R&D Systems, Minneapolis, MN) for IL-4, IL-10, IL-17, IFN-ɣ, IL-22, IL-21, or by Th1/Th2 10-Plex and IL-17-Tissue Culture Kits (read by MSD Sector Imager 2400, Meso Scale Discovery, Gaithersburg, MD). Supernatants were harvested from primary cultures at day 3 (day 2 for T-cell lines/clones). Production of cytokines was also analyzed by flow cytometry after culture of PBMCs with LL37/LL37-peptides for 6-7 days. Cells were activated with phorbol myristate acetate (PMA) (Sigma)/Ionomicin (Calbiochem) (PMA+Iono) for 4 hours and secretion blocked with GolgiStop (BD Biosciences). T-cell clones were cultured with HLA-matched-B-LCLs either unpulsed or pulsed with cognate peptide-antigen overnight. Cells were permeabilized and fixed by using the Intracellular fixation/permeabilizazion buffer set (eBiosciences). T-cells were gated on CD3/CD4 or CD3/CD8 expression and cytokine-staining analyzed on $BrdU^{+}$ cells. T-cells IL-17 production was also revealed by "Cytokine secretion assays kits" (MACS, Miltenyi), according to the Manufactur's instructions in primary bulk cultures set-up with LL37 or control REV peptide for 7 days (hrIL-2 was added at day 3 of culture, 20U/mL) and then re-stimulated with autologous irradiated PBMCs and peptide antigen at 10 ɣg/mL (LL37) over two hours.

Peptide-MHC-tetramer staining of T-cell lines/clones and blood T-cells

Peptide-MHC-tetramers (Table S4) were from ProImmune, (UK), TC Metrix, (CH), Benaroya, (USA) and ImmunAware (Denmark). Staining was performed for 40 minutes at 37°C, followed by staining for CD4 (4°C, 20 minutes). Before flow cytometry-acquisition, cells were labelled with PercP-conjugated 7-AAD to exclude dead cells.

LL37 specific T-cell help for production of autoantibodies

We cultured patient PBMCs with LL37 or control antigens and low doses of hrIL-2 (added at day 6, 10U/mL), and we detected appearance of autoantibodies in the culture supernatants by ELISA at various time points (from 7-to 10 days and, when needed, after a second stimulation with autologous PBMCs and antigen). In parallel, we monitored appearance of markers typical of B-cells/plasma cells (CD20, CD27, CD38, CD138) in the $CD3^{neg}$ cells by flow cytometry.

Immunohistochemistry and laser scanner confocal microscopy (LSM)

We performed staining on 6-mm paraffin or frozen sections of human lupus erythematosus skin specimens obtained from CHUV, Dept. of Dermatology. Staining with anti-cit-LL37 Mabs was performed using the antibodies Mab142 and Mab137 at 1:40 dilution, followed by incubation with a mouse antibody HRP (Abcam). HRP and DAB (DAKO) were used to develop the color. Nuclear staining was performed with Mayer's haematoxylin.

To visualize NETs, we seeded neutrophils on coverslips pre-treated with polylysine and stimulated them with various stimuli to induce release of the NETs. After 3 hours, cells were gently washed and stained with anti-CD15-PE or biotinylated polyclonal rabbit anti-LL37 antibodies (Innovagen), followed by addition of AlexaFluor 488-streptavidin. Frozen or paraffin-included SLE renal biopsies, obtained from Policlinico Umberto I, Rome, IT or CHUV Lausanne, CH, were stained, after saturation with blocking buffer (PBS, 0.05% tween 20, 4% BSA) for 1 hour at room temperature. Bopsies in paraffin were stained after de-paraffination in xilene (5 minutes, two times), followed by passages in: absolute ethanol (3 minutes), 95% ethanol in water (3 minutes), 80% ethanol in water (3 minutes), 70% ethanol in water (3 minutes), and antigen retrieval (5 minutes at

95°C in 10 mM sodium citrate, pH 6.0). Antibodies (Mab142 or Mab137) were added at 1:50 dilution (all diluited in blocking buffer) (isotype control was added at 1:100 concentration) for 1 hour at room temperature in a humidified chamber. Anti-C9 (Atlas antibodies, Sweden) was added at 1:50 dilution (all antibody dilutions were in blocking buffer). Slides were washed three times for 3 minutes under agitation with PBS 0.1% tween 20 and incubated with donkey anti-mouse or anti-rabbit secondary antibody or mouse anti-goat antibody, conjugated with AlexaFluor 647, Alexa Fluor 568 or 594 or AlexaFluor 488, depending on the combinations of markers analyzed (in humidified chamber). Slides were washed again, and mounted in Prolong Gold anti-fade media containing a DNA dye (DAPI) (Molecular Probes), before analysis with a confocal microscope, objectives 20x, or 40x and 60x in oil immersion. Images were taken by a FV1000 confocal microscope (Olympus, Tokyo, Japan), using a Olympus planapo objective 40x or 60x oil A.N. 1,42. Excitation light was obtained by a Laser Dapi 408 nm for DAPI, an Argon Ion Laser (488 nm) for FITC (Alexa 488), a Diode Laser HeNe (561 nm) for Alexa 568, and a Red Diode Laser (638 nm) for Alexa 647. DAPI emission was recorded from 415 to 485 nm, FITC emission was recorded from 495 to 550 nm, Alexa 568 from 583 to 628 nm and Alexa 647, from 634 to 750 nm. Images recorded had an optical thickness of 0.3 μm.

For quantification of LL37 and cit-LL37 in renal/skin biopsies, we counted the specific staining spots in at least 3 sections from each patient, by using the ImageJ64 software. For comparison we also counted the staining in HD skin, as background staining. To estimate the percent of co-localization between IgG and LL37 staining we measured, in three sections for each patient, the total IgG or LL37 staining in single color images and than on merge images we assessed the spots that indicates the co-localization IgG/LL37.

Reactivity of mouse anti-LL37 monoclonal antibodies (Mab137 and Mab142)

Biotinylated native LL37, cit-SCR LL37, SCR LL37, cit-LL37, and the other citrullinated and non-citrullinated control antigenic peptides (also biotinylated) were diluted at 2-to-5 μg/mL and added to the wells over night at 4°C. Excess unbound peptides were washed away with wash buffer (PBS

0.05% tween-20, 0.1% BSA). Antibody supernatants were added at dilution of 1:10, 1:20, 1:40, 1:100, 1:1000, in wash buffer, and incubated for 30 minutes at room temperature. After further three washing, a secondary anti-mouse antibody HRP conjugated (1:1000, Sigma) was added to the wells for 30 minutes and revealed as above.

B-LCLs

B-LCLs from SLE, CLE patients or HD were generated by incubating $2x10^5$-10^6 PBMCs with Epstein-Barr virus (EBV) from Marmoset lymphoblastoid-cell-line B95-8, in the presence of cyclosporine A (1 μg/mL). B-LCLs were derived in the same way from LL37-specific bulk cultures containing B-cells (at day 15 of culture). B-LCLs were cultured in T25 flasks (Corning Incorporated, Corning, NY) in RPMI 1640, 10% heat-inactivated Fetal Calf Serum, 2mM L-glutamine, 10 U/mL penicillin, and 100μg/mL streptomycin (B-cell-medium), and regularly passaged. For culture of B-LCLs to produce supernatants for funtional tests, cells were seaded at a fixed concentration and harvested at the same concentration ($1x10^6$/mL).

Statistical analyses

Differences between mean values were assessed by Student't test for single comparison. We used Student's t-test for paired samples (or Wilcoxon's matched-paires signed rank test, in case of low patients' sample size) to compare responses to antigens and control antigens in the same donor. We used Student's t-test for unpaired samples (or Mann-Whitney test for a lower number of patients' samples), for comparison of T-cell and antibody responses between groups of patients and HD. Statistical significance was set at $P< 0.05$. Correlation analyses were performed by Spearman's rank-correlation test. The frequency of T-cell responses to both native LL37 and cit-LL37 in SLE patients (with or without malar rash) was evaluated by Chi-square test. Statistical significance was set at $p < 0.05$. Data were analyzed by GraphPad Prism 7.0, or SPSS software.
